# Multifractal characterization of meteorological to agricultural drought propagation over India

**DOI:** 10.1038/s41598-024-68534-0

**Published:** 2024-08-14

**Authors:** Akshay Bajirao Pachore, Renji Remesan, Rohini Kumar

**Affiliations:** 1 School of Water Resources, Indian Institute of Technology Kharagpur, Kharagpur, 721 302 India; 2 Department of Computational HydroSystem (CHS), Helmholtz Centre for Environmental Research — UFZ, Permoserstraße 15, 04318 Leipzig, Germany

**Keywords:** Drought propagation time, SPI-n, SSMI-1, Meteorological drought, Agricultural drought, Multifractality, MF-DFA, Climate sciences, Environmental sciences, Environmental social sciences, Hydrology, Engineering

## Abstract

Agricultural drought affects the regional food security and thus understanding how meteorological drought propagates to agricultural drought is crucial. This study examines the temporal scaling trends of meteorological and agricultural drought data over 34 Indian meteorological sub-divisions from 1981 to 2020. A maximum Pearson's correlation coefficient (MPCC) derived between multiscale Standardised Precipitation Index (SPI) and monthly Standardised Soil Moisture Index (SSMI) time series was used to assess the seasonal as well as annual drought propagation time (DPT). The multifractal characteristics of the SPI time series at a time scale chosen from propagation analysis as well as the SSMI-1 time series were further examined using Multifractal Detrended Fluctuation Analysis (MF-DFA). Results reveal longer average annual DPT in arid and semi-arid regions like Saurashtra and Kutch (~ 6 months), Madhya Maharashtra (~ 5 months), and Western Rajasthan (~ 6 months), whereas, humid regions like Arunachal Pradesh, Assam and Meghalaya, and Kerala exhibit shorter DPT (~ 2 months). The Hurst Index values greater/less than 0.5 indicates the existence of long/short-term persistence (LTP/STP) in the SPI and SSMI time series. The results of our study highlights the inherent connection among drought propagation time, multifractality, and regional climate variations, and offers insights to enhance drought prediction systems in India.

## Introduction

According to a global assessment report on drought^1^, drought is a complex natural disaster and different climatic zones have different definitions of what a drought is. Drought events have different consequences and impacts on industrial, agricultural, and other water-use sectors of the region and these are difficult to compare throughout the space and time, attributable to the more regional character of this disaster. A different set of indices has been developed in the past to reflect water storage stress in different terrestrial compartments^2^. A drought event is recognized when one or more indicators fall below a particular threshold for a set amount of time. The use of a number of indicators allows for the study of how drought spreads through the hydrological cycle as well as the monitoring of its impact on different aspects of the economy and the environment^1,3,4^. By examining the characteristics and implications of drought, there are four major classifications: meteorological, agricultural, hydrological, and socioeconomic droughts^5^. Prolonged precipitation deficiency causing meteorological drought is generally considered a tipping point for water stress to propagate via the hydrological cycle^3,6,7^. When the increased actual evaporation demand co-occurred with the reduced precipitation, then, it will result in the depletion of soil moisture content. This reduced soil moisture in the crop root zone depth will further affect crop growth and yield causing agricultural drought in the region^8^. Previous studies have considered the soil moisture as a proxy for agricultural drought by limiting the depth of the soil moisture data to the crop root zone^9–11^. The soil moisture stress may not always reflect in the vegetation stress due to the short drought duration and hence researchers are using soil moisture drought and vegetation drought terms separately to avoid confusion, nevertheless both are the proxies for the agricultural drought assessment^12^. India, a country heavily reliant on agriculture and monsoon, is particularly vulnerable to the adverse effects of drought and some studies have highlighted how it is severely affecting its agriculture, economy, and society^13,14^. Furthermore, studying the drought propagation from meteorological to agricultural drought is crucial all over the world as it plays a decisive role in the creation of early warning systems through the analysis of gathered data and application of models^8^.

The transmission of drought conditions from a meteorological to an agricultural one has been studied in past studies^3,4,10,15,16^. Most of the drought propagation studies have used SPI (Standardized Precipitation Index), and SPEI (Standardized Precipitation Evapotranspiration Index) as meteorological drought indicators, whereas, SSMI (Standardized Soil Moisture Index), SMP (Soil Moisture Percentile), and NDVI (Normalized Difference Vegetation Index) for agricultural drought^10,15,17,18^. Previous studies have examined the meteorological to agricultural (soil moisture) drought propagation using a maximum Pearson’s correlation coefficient (MPCC) based approach to quantify the drought propagation time (DPT) and lag time scale of the meteorological drought^16,19–21^. There are very few regional studies in India that concentrate on the examination of the propagation mechanism that triggers agricultural drought. The recent study^11^, has examined the propagation of drought conditions from meteorological to agricultural realms over India. Large-scale climatic oscillations and local hydro-meteorological variables were used in the computation of the Standardized Precipitation Evapotranspiration Index (SPEI) and Standardised Soil Moisture Index (SSMI). Many authors^22,23^ have investigated the relationship of hydrological drought with meteorological one in the Indian context, but the analysis of agricultural drought propagation mechanism is rarely studied.

From the system engineering point of view, droughts are one of many natural phenomena that may be seen as complex systems^24,25^. One of the key ideas to be used in the understanding of complex systems has been the theory of chaos and fractals^26–28^. It is beneficial to have the knowledge of long-range and short-range power-law correlations in the variables like precipitation, river discharge, and related extreme hydro-meteorological events as well because it allows for improved modeling and forecasting of these variables and associated event characteristics^29^. The Detrended Fluctuation Analysis (DFA)^30^, has been utilized in past for the examination of long-range correlations and fractal characteristics in non-stationary hydrological time series of variables like river discharge, precipitation, and meteorological drought index^31–33^. DFA is applicable to monofractal time series having a single fractal exponent. A single exponent is sometimes insufficient to completely characterize the time series having multifractal characteristics and requires a continuous exponent spectrum for an accurate description. To assess these multifractal time series and to overcome the limitations of (monofractal) DFA, the MF-DFA (multifractal detrended fluctuation analysis) method was proposed^34^. MF-DFA has been applied by many scholars^34^ to explain the dynamic and complex fluctuation characteristics of the geophysical time series.

Multifractal applications to drought are rarely studied globally as well as for the Indian conditions. The majority of earlier research has individually focused on drought propagation and multifractality but has not studied them in an integrated approach. Such an approach would enable better identification of areas having lower predictability along with a higher risk of prolonged droughts and help allocate resources for timely mitigation and responses. It is therefore vital to analyse the relation of DPT with multifractality—an indicator of predictability—and how it varies spatially across different hydroclimatic conditions. The present study aims to examine the connections between drought propagation and persistence (long and short memory) as well as multifractal dimensions under climatic regions of India focusing on all 34 meteorological sub-divisions. To achieve the above-mentioned goals of the study, the following particular objectives were formulated, (1) to inspect the links between meteorological and agricultural drought using standardized quantitative drought indices (SPI and SSMI) and to quantify the seasonal as well as annual DPT; (2) to analyse the multifractality using MF-DFA in the SPI time series at the time scale selected from drought propagation study and SSMI-1 time series for different meteorological sub-divisions across India; (3) to study the interplay between the drought propagation mechanism and multifractality along with its spatial variation over different meteorological sub-divisions of India. Overall, our study on the joint analysis of multifractality and drought propagation aims toward providing a holistic understanding of drought propagation behaviour under local-scale impacts and regional-scale influences.

## Materials and methods

### Study area and data

**Figure 1:**
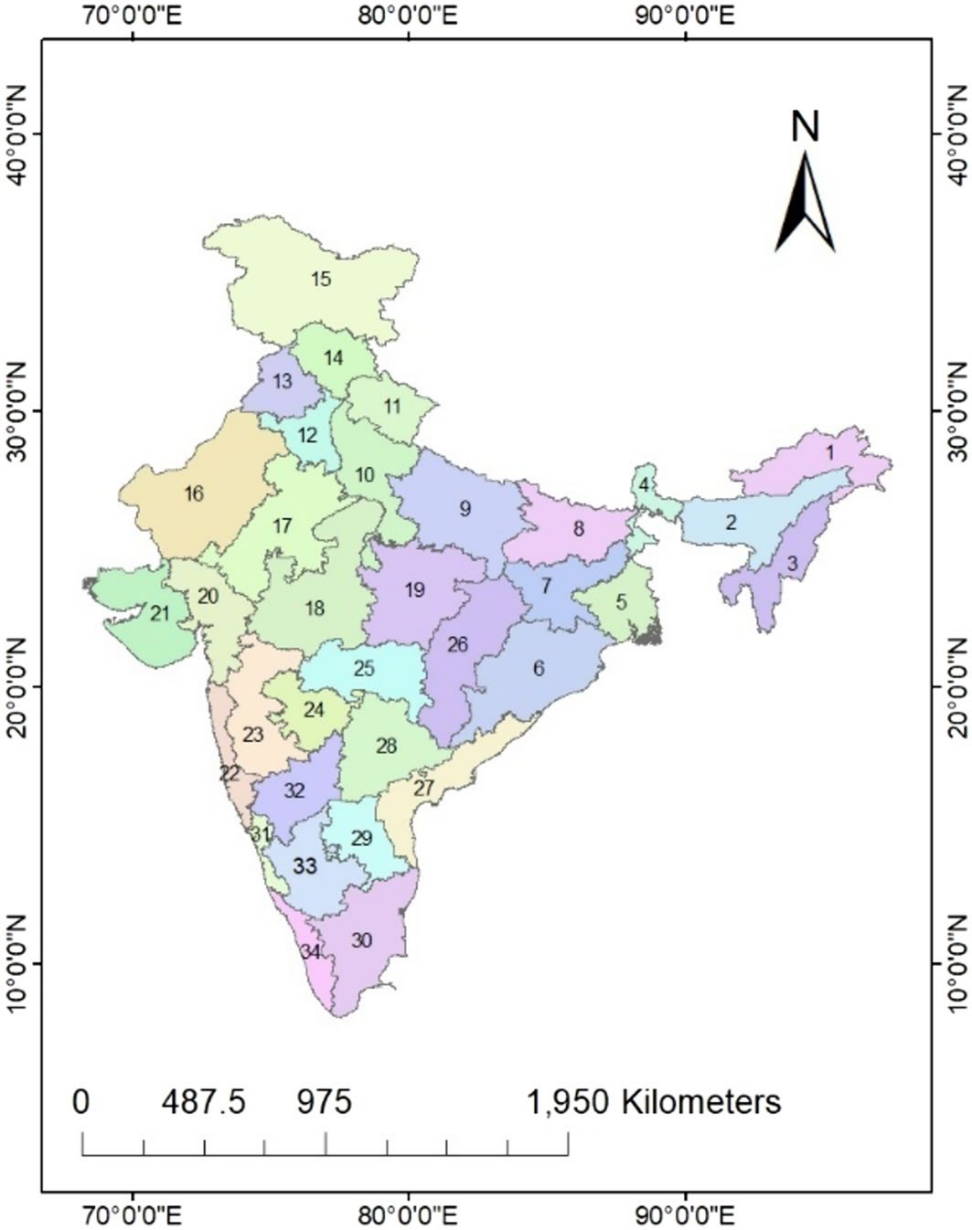
Study area map showing the location of all 34 meteorological sub-divisions of India [1.Arunachal Pradesh (AP); 2. Assam & Meghalaya (AM); 3. Nagaland, Manipur, Mizoram &Tripura (NMMT); 4. Sikkim (SKK); 5. Gangetic West Bengal (GWB); 6. Orissa (OR); 7. Jharkhand (JKH); 8. Bihar (B); 9. East Uttar Pradesh (EUP); 10. West Uttar Pradesh (WUP); 11. Uttarakhand (UK); 12.Haryana, Delhi, & Chandigarh (HDC); 13. Punjab (PJ); 14. Himachal Pradesh (HP); 15. Jammu & Kashmir (JK); 16.West Rajasthan (WRJ); 17.East Rajasthan (ERJ); 18.West Madhya Pradesh (WMP); 19. East Madhya Pradesh (EMP); 20.Gujarat (GJ); 21. Saurashtra & Kutch (SK); 22. Konkan & Goa (KG); 23. Madhya Maharashtra (MMRH); 24.Marathwada (MRTH); 25.Vidarbha (VDH); 26. Chattisgarh (CG); 27. Coastal Andhra Pradesh (CAP); 28. Telangana (TL); 29.Rayalaseema (RS); 30. Tamil Nadu (TN) 31. Coastal Karnataka (CK); 32. North Interior Karnataka (NIK); 33. South Interior Karnataka (SIK); 34. Kerala (KL)]. (The Figure was generated by ArcGIS 10.3 software, https://www.esri.com/en-us/arcgis/products/arcgis-desktop/resources).

Mainland India has been classified into 34 meteorological sub-divisions (as shown in Fig. 1) by the India Meteorological Department (IMD) based on rainfall homogeneity and similarity in climate^35^. The present study uses the IMD gridded (0.25° × 0.25°) precipitation dataset which is available at a daily time step. The daily precipitation data is aggregated to the monthly time step, and the long-term area-averaged estimates are prepared for all the 34 meteorological sub-divisions of India from 1981 till 2020 (40 years) which is further used as input for the computation of SPI.

Due to the unavailability of spatio-temporally consistent in-situ soil moisture observations for India, the present study uses the ERA5 soil moisture data to get the long-term time series^36,37^. ERA5 provides long-term data on different land variables from 1950 till the present day at 0.25° × 0.25° spatial and hourly temporal resolution. Reanalysis employs the physical model to merge modeled simulations with global observations to create a globally comprehensive and consistent dataset using data assimilation approaches^36,38,39^. The data assimilation algorithm has been incorporated in ERA5 for soil moisture simulations from 1979 onwards^40^, hence the present study is limited to the period of 40 years which starts from 1981 onwards till 2020. ERA5 soil moisture is available at different depths which are layer 1 (0–7 cm), layer 2 (7–28 cm), layer 3 (28–100 cm), and layer 4 (100–289 cm). For the present study, soil moisture data is extracted for the depth of 60 cm as this is the crop root zone depth for the majority of the Indian crops^14,41,42^ and converted from hourly to daily scale (See Fig. S1 of the supplementary material) by following the approach used in the past literature^43^. To study the drought propagation and associated multifractality, standardized drought indices (SPI and SSMI) were computed using the standardized precipitation and soil moisture time series^9,44^. Additionally, NDVI time series data is generated from Advanced Very High-Resolution Radiometer (AVHRR) GIMMS, 3rd generation^45^, to demonstrate the correlation between vegetation health (NDVI) and rainfall anomaly (SPI). The NDVI time series is prepared from 1982 till 2020. GIMMS NDVI is available from 1982 to 2015 and the remaining is extrapolated till 2020 using the linear regression relationship between GIMMS NDVI and MODIS NDVI for the overlapping period (2001–2015)^46^. The standardized anomaly of NDVI is calculated from the prepared NDVI time series, which further utilized to build correlation with SPI and also to analyze the respective multifractal features for the period from 1982 till 2020.

### Methodology

**Figure 2:**
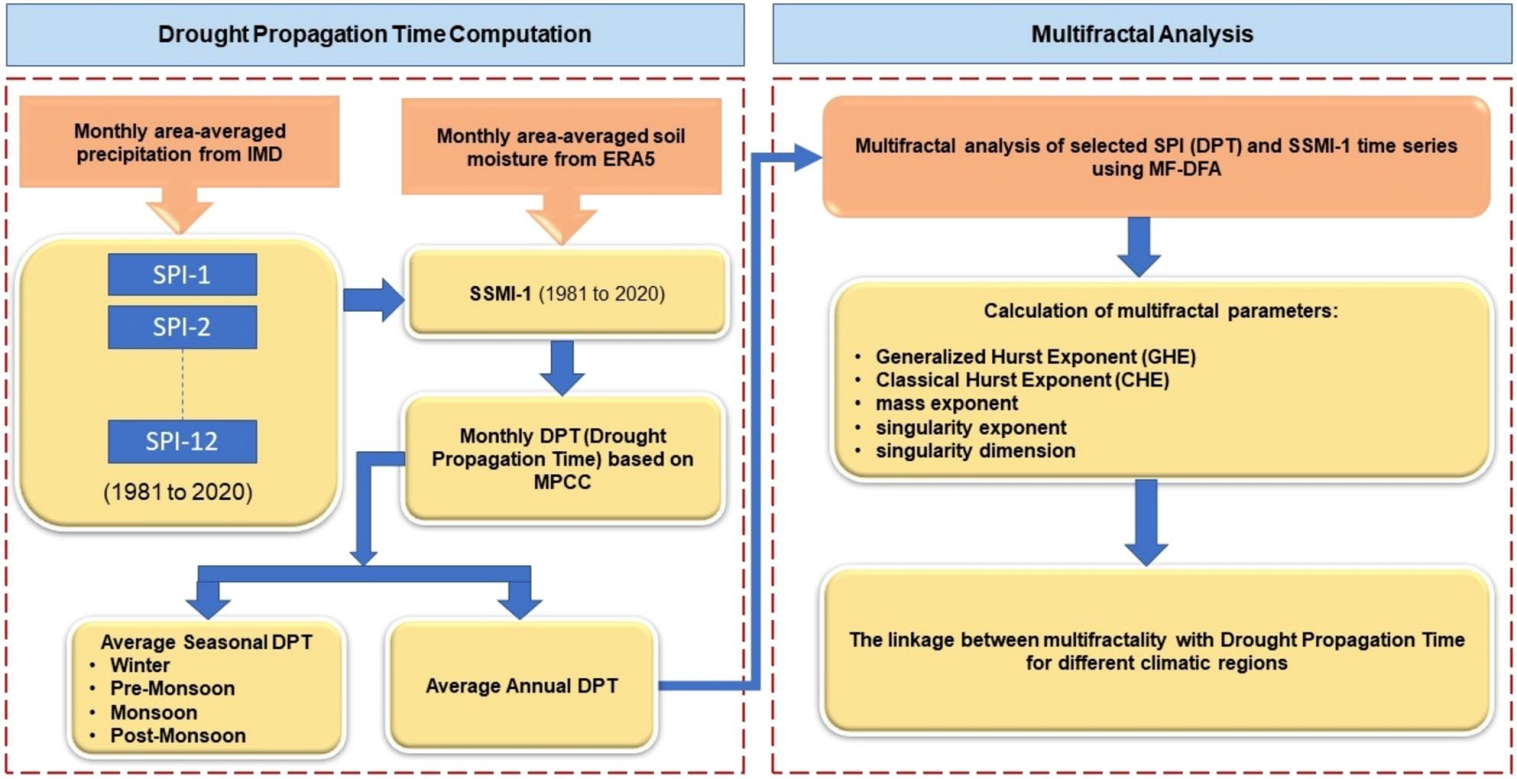
Methodological framework to study the interconnection between drought propagation and multifractality over India.

The present study aims to analyze the interplay between the persistence, multifractality, and propagation of drought as explained in the flowchart (see Fig. 2). To achieve this, first, propagation time from meteorological to agricultural drought is computed by applying the Pearson’s correlation coefficient (PCC) method. PCC between multiscale SPI (SPI-n) and monthly SSMI-1 was calculated to identify the accumulation month “n” with maximum PCC which was then treated as DPT. Further, the multifractal detrended fluctuation analysis (MF-DFA) method was utilized to study the multifractal properties of the selected SPI time series from the MPCC results and SSMI-1 time series for each of the 34 meteorological sub-divisions of India.

The following section describes the details of drought index computation, Pearson’s correlation coefficient (PCC), and MF-DFA methods used in the current study.

#### Computation of drought indices (SPI and SSMI):

The standardized precipitation index (SPI;^47^) is commonly accepted for meteorological drought assessment and is also recommended by WMO^48^ and it is calculated at multiple aggregation time steps starting from the shortest (say one month) to largest (e.g., 72 months) aggregation level depending on the need of water resources management and policy related implementations at different locations. SPI is calculated as a standardized anomaly of the precipitation at different aggregation time scale from the long term mean value and follows the Eq. (1).

1 $$ {\text{SPI}}\, = \,\frac{{X_{i} - \overline{X}}}{\sigma } $$

where $X_{i}$, is the monthly precipitation at different aggregation time scale and $\overline{X}$ is the long-term mean value of the precipitation. Numerator of the Eq. (1) gives the anomaly value which is divided by the standard deviation (σ).

As SPI is a measure of the transformed probability of precipitation at a given aggregation time scale, when computed at moderate time scales (3-6 months), it may reflect variability of root-zone soil water content, when computed at longer time scales (12–24 months) then may reflect the behaviour of groundwater and streamflow components of water resources^2,5,49,50^. For the computation of SPI, long-term precipitation data is fitted with the gamma distribution function (see Eq. 2).

2 $$ g\left( {x_{k} } \right) = \frac{1}{{\beta^{\alpha } \tau \left( \alpha \right)}} x_{k}^{\alpha - 1} e^{{{\raise0.7ex\hbox{${x_{k} }$} \!\mathord{\left/ {\vphantom {{x_{k} } \beta }}\right.\kern-0pt} \!\lower0.7ex\hbox{$\beta $}}}} \,\,\,{\text{for}}\,\,\,{\text{x}}_{{\text{k}}} \, > \,0 $$

where α is a shape factor, β is a scale factor, and x_k_ is the amount of precipitation over k consecutive months.

The data fitted to the gamma distribution function is further utilized to calculate the cumulative distribution function (CDF) to take care of zero values of precipitation, which is not taken into account by the gamma distribution. In the last step, the inverse of standard normal CDF is calculated to get the SPI values. Readers can refer to^47^ for the details of the step-by-step procedure for the computation of SPI. Previously, many researchers have used the Standardized Soil Moisture Index at a monthly timescale (SSMI-1) to characterize agricultural drought^51–53^. The SSMI is used at a one-month timescale and aggregation can be avoided, as the current month’s soil moisture represents the past information because of the accumulating nature^54^. The computation algorithm for the SSMI is the same as that of SPI and it uses the monthly averaged soil moisture value as input data instead of precipitation (see Eq. 1). Agricultural drought properties (duration and severity) based on the SSMI-1 time series were computed to see the variation over different meteorological sub-divisions of India.

#### Correlation-based drought propagation time

Pearson’s correlation coefficient (PCC)-based approach has been tested by many scholars in the past^10,16,19^ to identify the lag time or DPT. PCC is generally used to quantify the linear relationship between the time series data of two variables and is calculated using Eq. (3).

3 $$ R = \frac{{\sum \left( {x_{i} - \overline{x}} \right)\left( {y_{i} - \overline{y}} \right)}}{{\sum \left( {x_{i} - \overline{x}} \right)^{2} \sum \left( {y_{i} - \overline{y}} \right)^{2} }} $$

where R is the Pearson’s correlation coefficient (PCC) between the variable x and y, x_i_ and y_i_ are the values of the x and y variables in the sample, $\overline{x}$ and $\overline{y}$ are the mean values of the x and y variables in the sample. Here, x corresponds to SPI-n (n = 1, 2, 3, 4,…12) and y corresponds to the monthly SSMI.

The PCC-based method was applied to calculate the propagation time between two drought types. PCC between SPI-n (n = 1, 2, 3,…12) and monthly SSMI was calculated, and the SPI time scale having maximum PCC (MPCC) value was treated as DPT for that region. The MPCC-based DPT was calculated for each season i.e., winter (Jan–Feb), pre-monsoon (Mar–May), monsoon (June–Sept), and post-monsoon (Oct–Dec), which were further used to get the average annual DPT for all 34 meteorological sub-divisions of India. MPCC for each region was calculated between multiscale SPI and monthly SSMI as shown in Eq,. (4).

4 $$ R_{n} = {\text{Corr}}\left( {{\text{SPI}} - {\text{n}},{\text{ SSMI}} - 1} \right) $$

here $R_{n}$ is the maximum Pearson’s correlation coefficient (MPCC) for the n^th^ month SPI with the monthly SSMI at a 5% significance level (*p* < 0.05). Further, various seasonal propagation times for all meteorological sub-divisions are calculated from the monthly values of DPT after performing the significance test.

#### Multifractal Detrended Fluctuation Analysis (MF-DFA)

MF-DFA is the further development of monofractal DFA that has been applied in the past^34,55,56^ to describe the multifractal characteristics in the hydrologic time series data. MF-DFA is used to detect the autocorrelation and multifractality in the meteorological and agricultural drought index time series as explained below. In the first step, profile D(i) is determined, which is calculated using Eq. (5) given below,

5 $$ D\left( i \right) = \mathop \sum \limits_{k = 1}^{i} \left[ {y_{k} - \overline{y}} \right] $$

here profile D(i) is the cumulative deviation of the individual data points of the drought index time series $y_{k}$ from the long-term mean $\overline{y}$. Where i varies from 1, 2, 3,…,N, and N is the length of the time series.

After generating the profile data, it is further segmented into N_s_ = int(N/s) the number of divisions (int corresponds to integer), where ‘s’ is the length of individual division, traditionally referred to as scale. As the length (N) of time series may not be the integer multiple of scale (s), hence, it is possible that a smaller portion at the end of the series may get omitted. To take care of this omitted part, the same process is repeated starting from the opposite end, resulting in 2Ns segments.

In the next step, the method of least of the square is employed to estimate the local trend of each segment using the Eqs. (6) and (7)

6 $$ F^{2} \left( {s,v} \right) = \frac{1}{s} \mathop \sum \limits_{i = 1}^{s} \{ D[\left( {v - 1} \right)s + i\widehat{{D_{v } }}\left( {\text{i}} \right)\}^{{2}} $$

where v is the segment and varies from 1, 2,…till N_s_ and

7 $$ F^{2} \left( {s,v} \right) = \frac{1}{s} \mathop \sum \limits_{i = 1}^{s} \left\{ {D[N - \left( {v - N_{s} } \right)s + i] - \widehat{{D_{v } }}\left( {\text{i}} \right)} \right\}^{{2}} $$

$$ {\text{v }} = {\text{ N}}_{{\text{s}}} ,{\text{ N}}_{{\text{s}}} + {1}, \ldots ,{\text{ 2N}}_{{\text{s}}} $$

In the above-mentioned equation, the fitting polynomial of any suitable order in segment i is represented here by D_v_ (i).

After calculating the $F^{2} \left( {s,v} \right)$ (variance), the fluctuation function of qth order is calculated using Eq. (8)

8 $$ F_{q} \left( {\text{s}} \right)\, = \,\left\{ {\frac{1}{{2N_{s} }}\mathop \sum \limits_{v = 1}^{{2N_{s} }} [F^{2} \left( {s,v} \right)]^{{{\text{q}}/{2}}} } \right\}^{{{1}/{\text{q}}}} $$

here q can take any real value except zero and if fluctuation function is to be calculated for q = 0 then the following Eq. (9) is used.

9 $$ F_{q} \left( {\text{s}} \right)\, = \,{\text{exp}}\left\{ {\frac{1}{{4N_{s} }}\mathop \sum \limits_{v = 1}^{{2N_{s} }} ln[F^{2} \left( {s,v} \right)]} \right\} $$

Scaling characteristics of the fluctuation function for different values of moment order ‘q’ can be determined with the help of a log–log plot of Fq(s) vs s. If the time series shows power law behavior then it follows the relation F_q_(s)–s^H(q)^, where, H(q) is the Generalized Hurst Exponent (GHE). If the time series is stationary then, 0 $$< $$ H (q = 2) $$<$$ 1, is similar to a classical Hurst exponent, also known as Hurst index; H (q = 2). Whereas, for non-stationary time series data, H (q = 2) > 1, and the relation between GHE and classical hurst exponent will be modified to H(q) = H(q = 2) − 1. When the time series is uncorrelated then, H(q = 2), also known as the Hurst exponent or Hurst index is equal to 0.5. Long range memory or correlation or persistence in the time series is denoted by the range of Hurst exponent (0.5–1), whereas, short-range memory or antipersistence nature of time series is defined when Hurst exponent lies between 0 and 0.5^55,56^.

Long Term Persistence (LTP) means time series has positive autocorrelation i.e., current observation has an influence on observation in the next time step. For monofractal time series, GHE is independent of moment order q, but this is not the case in multifractal time series since small and large fluctuations have varying occurrence patterns. To quantify the effect of small and large fluctuations, both positive and negative values of moment order ‘q’ are considered. The scaling behavior of the segments with large fluctuations can be defined using positive values of ‘q’, whereas, negative values of q can be used for the quantification of the scaling behaviour of the segments with small fluctuations. When employing the MF-DFA algorithm, some important considerations are the choice of polynomial, minimum and maximum scale bound, and scale size. The selection of these parameters is to be done by following the guidelines given in the past literature ^29,57,58^.

#### Assessment of multifractality strength

For assessing the strength and evidence of multifractality in the time series, different scaling exponents are derived, namely: mass exponent (tq), singularity exponent (hq), and singularity dimension (Dq) (see Eqs. 10, 11, and 12 below). The general procedure in literature^59^ is to convert the q-order Hurst exponent to the q-order mass exponent (t(q)) which is further used to calculate the singularity exponent (h(q)) and singularity dimension (D(q)).

10 $$ {\text{t}}\left( {\text{q}} \right) \, = {\text{ qH}}\left( {{\text{q}} = {2}} \right) \, {-}{ 1} $$

for non-stationary time series having a multifractal nature, t(q) will have a non-linear dependency on the qth order.

The singularity dimension (D(q)), which is connected to the mass exponent (t(q)) by a Legendre transform, is another approach to describe a multifractal strength in time series.

11 $$ {\text{h}}\left( {\text{q}} \right) \, = \frac{dt\left( q \right)}{{dq}} $$

and

12 $$ {\text{D}}\left( {\text{q}} \right) \, = {\text{ q h}}\left( {\text{q}} \right) \, {-}{\text{ t}}\left( {\text{q}} \right) $$

h(q) is the singularity strength or singularity exponent, whereas, D(q) is denoted as the singularity dimension which is a function of singularity exponent and mass exponent.

The degree/strength of multifractality of the time series is defined by the difference between maximum and minimum values of singularity exponent (hq_max_ − hq_min_). The multifractal spectrum’s shape is not necessarily symmetric and is similar to an inverted parabola. The q-order Hurst exponent can be levelled for positive or negative q's, respectively, causing the left or right truncation of the multifractal spectrum^57^.

## Results

### Spatial distribution of drought properties

**Figure 3:**
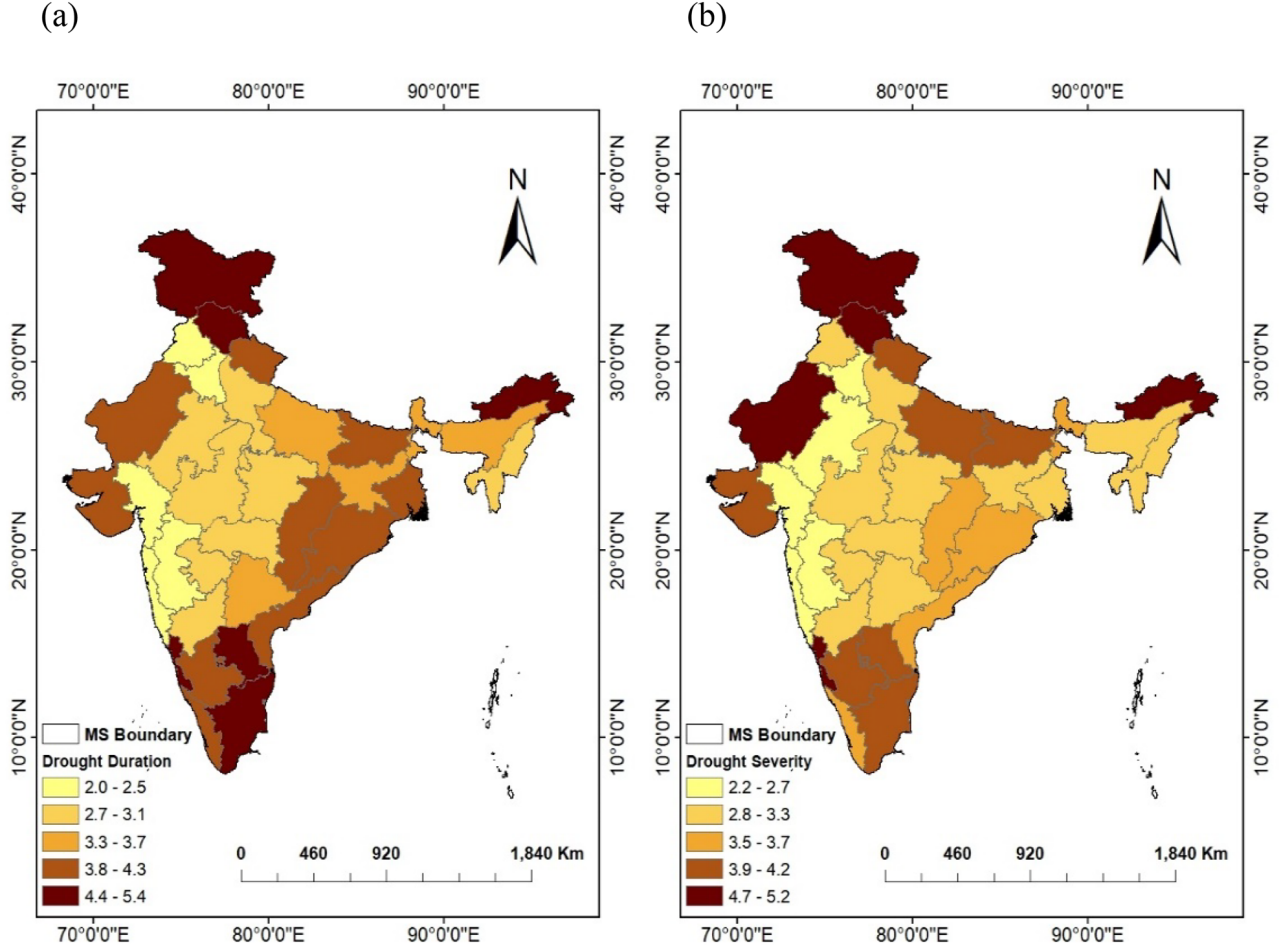
SSMI-1 based (**a**) average agricultural drought duration and (**b**) severity for the duration from 1981 till 2020 over 34 meteorological sub-divisions of India. (The figure was generated by ArcGIS 10.3 software, https://www.esri.com/en-us/arcgis/products/arcgis-desktop/resources).

The spatial distribution of agricultural drought properties i.e., average drought duration and severity based on SSMI-1 time series computed using ERA-5 soil moisture product over the various meteorological sub-divisions of India during the period of 1981–2020 (40 years) is given in Fig. 3a,b. The average drought duration over all meteorological sub-divisions is observed to be varying in the range of 2.0–5.4 months, whereas, the range of average drought severity is from 2.2 to 5.2. It is observed that about ~ 26 % of the meteorological sub-divisions shows the average drought duration (severity) of 2.5 months (2.66); ~ 38 % shows, 3.44 months (3.49); and ~ 35 % shows, 4.46 months (4.21). Drought properties (duration and severity), when assessed using SSMI-1 values, it is observed that a few northern regions, such as Jammu and Kashmir (5.40 and 4.91), and Himachal Pradesh (4.89 and 4.08); along with a few southern regions, such as Coastal Karnataka (4.70 and 4.68) and Tamilnadu (4.47 and 4.23) have shown higher values of average drought duration and severity. Also, most of the regions from central India (arid and semi-arid climate) such as Marathwada (3.07, 3.08), Vidharbha (2.97, 2.91), Eastern Madhya Pradesh (3.00, 3.26), Western Madhya Pradesh (3.00, 3.27) are showing the drought duration and severity values in the same range (see Fig. 3). Overall, agricultural drought characteristics shows the significant spatial variation over India, with the observation that arid and semi-arid regions from central India having the homogeneous pattern. Identical findings were reported in past^11^ showing a similar pattern for the spatial variation of the agricultural drought properties over India. It is noteworthy that there is synchrony between drought duration and severity over Indian regions, as locations with greater levels of drought duration also exhibit higher values of drought severity. The above made observations indicates that it is worth monitoring the drought propagation phenomenon from one form to another at a seasonal scale in order to detect linear seasonal drought propagation dynamics and its relationship over the regions with different climate types.

### Spatial and seasonal variability of drought propagation over India

Table 1 shows the results of DPT computed using MPCC values for SPI-n and SSMI-1 pairs for each region incorporating pixel-by-pixel comparison after consideration of paired statistical tests^60^. Based on MPCC values, the average annual DPT for all divisions (all India average) was observed to be 2.83 months while the shortest seasonal propagation time is associated with post-monsoon season (2.08 months). Table 1 also indicates that different lag values of SPI timescales correlate well with the SSMI-1 for different meteorological sub-divisions of India which explains how meteorological drought impacts agriculture in India. In the present analysis, DPT for each month (Jan–Dec) is inferred by analyzing the monthly PCC values of SPI-n and SSMI-1, and further, these monthly DPT were utilized to get seasonal average DPT and annual average DPT for each of 34 meteorological regions across India. DPT shows a significant variation across seasons and regions over India, with generally winter (3.43 months) and pre-monsoon (3.36 months) seasons associated with higher seasonal DPT (all India average) as compared to monsoon (2.53 months) and post-monsoon (2.08 months) seasons. From the seasonal aspect, it was observed that the pre-monsoon season is having higher range for DPT (1–9 months), whereas, winter (1–6 months), monsoon (1–6.5 months), and post-monsoon (1–4.33 months) seasons are having lower ranges relatively. Based on the PCC values, a very weak and insignificant correlation was observed between multiscale SPI and monthly SSMI for HP, NIK, SKK, and WUP regions during the winter season. Therefore, DPT cannot be adequately interpreted for these regions by studying the direct (linear PCC) connection between soil moisture and precipitation. From average annual DPT results, it is evident that humid regions like AP (1.75 months), GWB (1.50 months), and SKK (1.20 months) are associated with lower DPT, whereas, higher values were observed for arid regions like GJ (5.67 months), SK (5.64 months), and WRJ (5.73 months).

**Table 1:** Mean annual and seasonal DPT based on MPCC (p < 0.05: significance level of 5%) for meteorological sub-divisions of India.

Sr. No	Meteorological Sub-division	Average Seasonal DPT				Average annual DPT	SPI timescale for MF-DFA analysis
		Winter	Pre-Monsoon	Monsoon	Post-Monsoon		
1	Arunachal Pradesh (AP)	1.50	2.67	1.25	1.67	1.75	2
2	Assam and Meghalaya (AM)	4.00	2.00	1.25	1.67	2.00	2
3	Bihar (B)	5.00	3.67	2	1.50	4.88	5
4	Chattisgarh (CG)	2.50	2.33	2.00	1.67	2.10	2
5	Coastal Andhra Pradesh (CAP)	1.50	2.00	1.00	3.00	1.86	2
6	Coastal Karanataka (CK)	3.00	2.67	2.33	2.00	2.40	2
7	East Madhya Pradesh (EMP)	3.00	5.00	1.33	1.33	2.64	3
8	East Rajasthan (ERJ)	5.50	4.00	1.50	2.00	3.10	3
9	East Uttar Pradesh (EUP)	4.50	2.00	1.33	1.33	2.09	2
10	Gangetic West Bengal (GWB)	2.50	1.33	1.25	1.33	1.50	2
11	Gujrat (GJ)	5.50	8.00	5.75	3.33	5.67	6
12	Hariyana Delhi Chandigarh (HDC)	1.50	1.33	1.25	2.00	1.40	1
13	Himachal Pradesh (HP)	XXX	1.00	1.00	2.67	1.63	2
14	Jammu and Kashmir (JK)	3.00	4.67	4.75	3.00	4.00	4
15	Jharkhand (JHK)	4.50	1.67	1.00	1.33	2.00	2
16	Kerala (KL)	2.50	2.67	4.00	1.33	2.29	2
17	Konkan and Goa (KG)	5.50	5.33	1.50	2.00	3.25	3
18	Madhya Maharashtra (MMRH)	5.50	5.33	6.50	2.33	5.00	5
19	Marathwada (MRTH)	5.50	5.33	2.00	2.67	3.58	4
20	Nagaland, Manipur, Mizoram, and Tripura (NMMT)	4.50	5.67	1.50	2.00	3.17	3
21	North Interior Karnataka NIK)	XXX	1.33	6.50	1.50	3.67	4
22	Orissa (OR)	2.00	2.67	1.33	4.33	2.64	3
23	Punjab (PJ)	1.00	4.33	1.00	1.50	2.10	2
24	Rayalseema (RS)	1.00	3.00	1.00	1.50	1.70	2
25	Saurashtra and Kutch (SK)	5.50	8.67	4.67	3.67	5.64	6
26	South Interior Karnataka (SIK)	1.50	2.67	6.00	1.50	3.20	3
27	Sikkim (SKK)	XXX	1.00	1.00	1.50	1.20	1
28	Tamilnadu (TN)	1.50	3.00	3.00	1.67	2.30	2
29	Telangana (TL)	2.50	3.00	4.67	2.00	3.09	3
30	Uttarakhand (UK)	1.00	1.67	1.75	2.00	1.70	2
31	Vidharabha (VDH)	4.50	1.33	2.25	2.00	2.33	2
32	West Madhya Pradesh (WMP)	5.50	1.33	1.67	3.00	2.60	3
33	West Rajasthan (WRJ)	6.00	9.00	4.67	3.33	5.73	6
34	West Uttar Pradesh (WUP)	XXX	2.67	2.00	1.00	2.00	2

XXX denotes the insignificant correlations at 5% significance level.

Our analysis for the whole India average also showed that 1-month SSMI is the best correlated with SPIs at different lag timescales such as 3.43 months (winter season), 3.36 months (pre-monsoon season), 2.53 months (monsoon season), 2.08 months (post-monsoon season) respectively. The findings of previously done study^61^ indicate that employing the SPI with a lag time of three months is suitable for evaluating the influence of drought on crop growth. Our results provides support for this notion, as similar DPT range is reported for the SPI-n correlation with the SSMI-1 computed based on root zone soil moisture (RZSM) having significance from agricultural drought point of view. The findings of this investigation have demonstrated how the local climate has a major impact on how quickly meteorological conditions affect the agricultural areas based on RZSM data. Identical findings were reported in the past^62^, in a study conducted across China's many climatic zones.

### Multifractal characterization of meteorological and agricultural drought index time series involved in propagation mechanism

**Figure 4:**
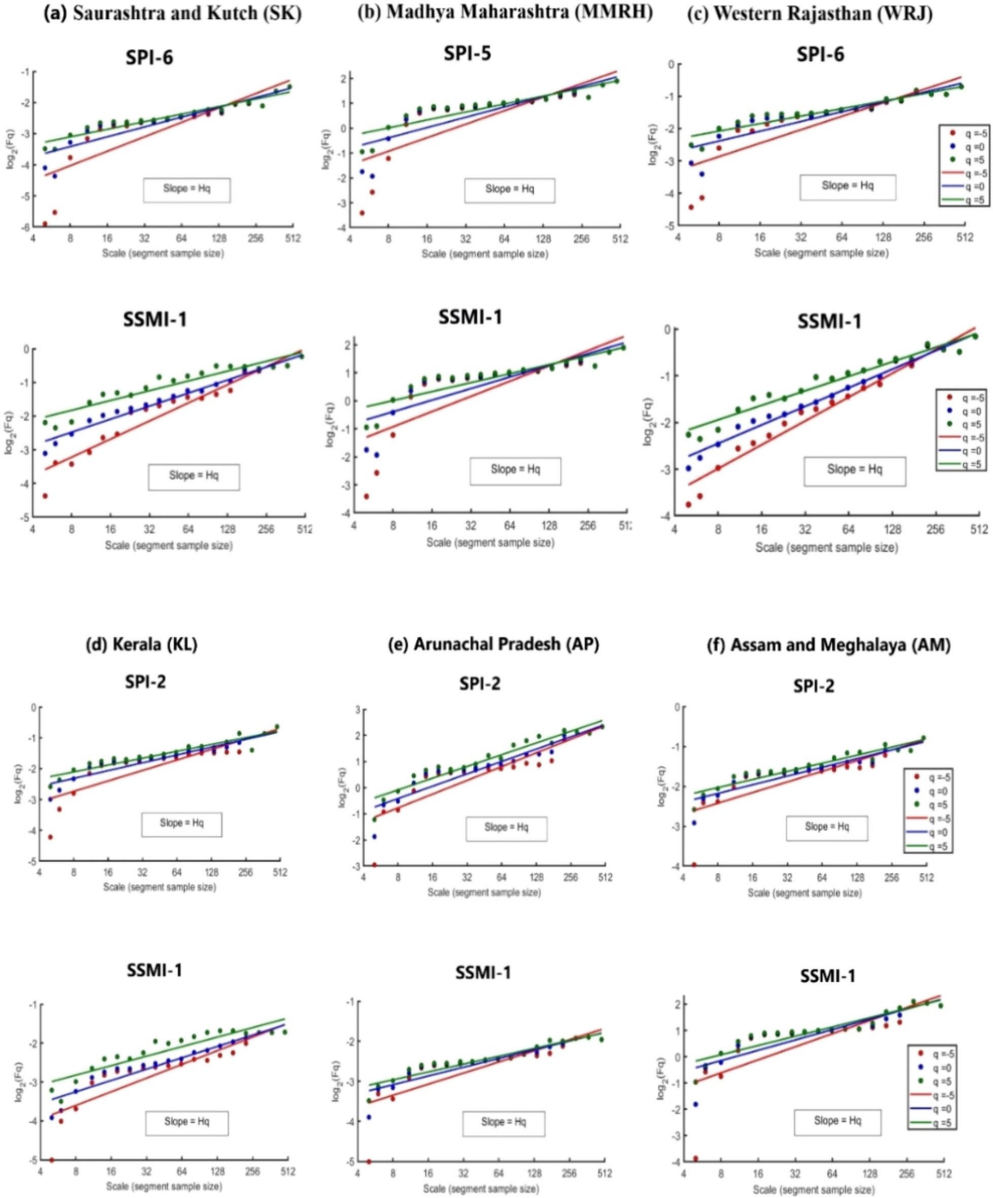
Fluctuation function plots for DPT-based SPI time series and SSMI-1 time series showing the evidence of multifractality for six representative meteorological sub-divisions.

The multifractal detrended fluctuation analysis (MF-DFA) algorithm, as described in sections “Multifractal Detrended Fluctuation Analysis (MF-DFA)” and “Assessment of multifractality strength” of the methodology, was utilized to extract information regarding the presence and extent of multifractality in the selected SPI time series, which were chosen based on a prior propagation study mentioned in previous paragraphs (see Table 1). Moreover, the obtained information concerning persistence and multifractality was compared with the DPT to identify potential interconnections. Multifractal characterization of SPI time series selected based on MPCC values from propagation study was analyzed. MF-DFA algorithm was applied to the SPI time series for 34 meteorological subdivisions of India by establishing a log-log relationship between Fq(s) (fluctuation function) and scale (s) (segment sizes). Figure 4 displays this analysis for the representative subdivisions. The representative regions were selected based on climatic conditions, three regions with humid climates (KL, AP, and AM) and three others with arid climates (SK, MMRH, and WRJ) to display the impact of climatic aridity on multifractality and drought propagation. Different values of slopes, termed as GHE (Generalized Hurst Exponent) was estimated for different moment order ‘q’, varying from − 5 to 5, which highlights the multifractal nature of the SPI time series.

**Figure 5:**
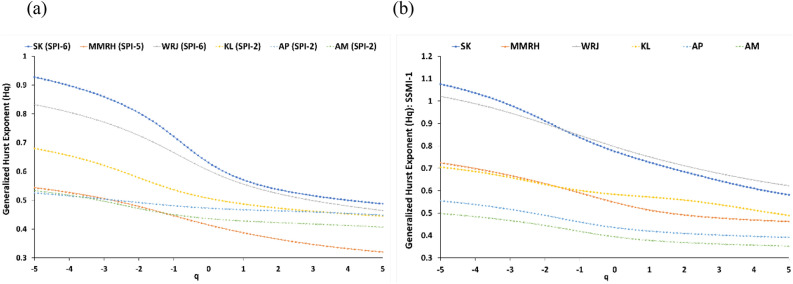
Generalized Hurst Exponent (GHE) plots for (**a**) SPI time series at different time scales (selected from DPT study) and (**b**) SSMI-1 time series for six representative meteorological sub-divisions.

The GHE variation against moment order q, as illustrated in Fig. 5, was systematically examined to ascertain the presence of multifractality, specifically in terms of non-linear dependence, across selected six representative regions of India. The logarithmic fluctuation function Fq(s) in these figures signifies the scaling behavior of the fluctuations in the SPI time series at different scales and the SSMI-1 time series for these regions. It provides information about the variations and self-similarity of the SPI and SSMI values across different temporal resolutions or segment sizes in each selected meteorological subdivision. The changes in the slope Hq values indicate that the severity and duration of drought events exhibit varying degrees of heterogeneity or intermittency across different scales in selected regions.

The summary of the MF-DFA results is presented in Fig. 5 for representative regions, namely Saurashtra and Kutch (SK), Madhya Maharashtra (MMRH), Western Rajasthan (WRJ), Kerala (KL), Arunachal Pradesh (AP), and Assam and Meghalaya (AM). For further multifractal characterization, the SPI timescales chosen corresponding to the DPT are: SPI-6 for SK, SPI-5 for MMRH, SPI-6 for WRJ, and, SPI-2 for AM, KL, and AP (see Fig. 5a), respectively, whereas, Fig. 5b shows the MF-DFA results of SSMI-1 time series for these regions.

The spread of GHE from moment order − 5 to 5 (∆Hq) for SPI-n and SSMI-1 was observed to be 0.44 and 0.49 for SK, 0.22 and 0.26 for MMRH, 0.37 and 0.40 for WRJ, 0.13 and 0.15 for AM, 0.23 and 0.22 for KL, 0.08 and 0.16 for AP respectively. Notably, regions with wet climates such as AM, KL, and AP exhibited the least variation of GHE with moment order (q), whereas arid regions like SK, MMRH, and WRJ displayed greater variation of GHE with q. A similar variation was observed for the DPT (refer to Table 2), with lower DPT values for AM (2.0 months), AP (~2 months), and KL (~2 months), and higher values for SK (~6 months), MMRH (5.00 months), and WRJ (~6 months) clearly indicating how the drought data exhibit distinct characteristics in terms of complexity and persistence for these regions with different climatic conditions. A similar finding indicating that the degree of multifractality increases with aggregation time scales of SPI and it is synchronous with the climate of the region is reported in the past literature^29^, but ignored the propagation mechanism by just focusing on meteorological drought index time series. These observations in Fig. 5a also highlight that regions characterized by arid or semi-arid climates exhibit higher multifractality and DPT, indicating a more intricate and diverse nature of the time series. Conversely, regions with wet/humid climates display lower levels of multifractality and DPT. From the results, it can be inferred that multifractality or complexity and DPT are synchronous with the climatic conditions of the respective regions. Similar findings were reported in a study from China^62^ indicating humid regions have low DPT (~3 months), whereas higher DPT in arid regions of China (up to 8 months). Similarly, in case of the agricultural drought time series, multifractal nature is observed to be in synchronous with the climate of the region which can be inferred from the GHE variation of SSMI-1 data in the selected six representative subdivisions (see Fig. 5b).

**Table 2:** Multifractal parameters and DPT for 34 meteorological sub-divisions of India.

Sr. No	Region	DPT in months	GHE range (∆Hq)		Spectrum width (∆hq)		Hurst Index (q = 2)	
			SPI-n	SSMI-1	SPI-n	SSMI-1	SPI-n	SSMI-1
1	AP	2	0.08	0.16	0.16	0.26	0.46	0.49
2	AM	2	0.13	0.15	0.22	0.23	0.42	0.44
3	B	5	0.19	0.26	0.28	0.39	0.32	0.48
4	CG	2	0.21	0.24	0.34	0.35	0.29	0.40
5	CAP	2	0.18	0.26	0.30	0.44	0.38	0.58
6	CK	2	0.11	0.17	0.21	0.21	0.36	0.46
7	EMP	3	0.25	0.32	0.38	0.47	0.32	0.45
8	ERJ	3	0.27	0.27	0.42	0.42	0.32	0.46
9	EUP	2	0.25	0.31	0.39	0.45	0.30	0.51
10	GWB	2	0.31	0.20	0.49	0.49	0.27	0.43
11	GJ	6	0.35	0.31	0.51	0.48	0.48	0.68
12	HDC	1	0.25	0.28	0.40	0.47	0.35	0.71
13	HP	2	0.30	0.34	0.46	0.53	0.52	0.73
14	J and K	4	0.27	0.28	0.48	0.42	0.75	0.41
15	JKH	2	0.21	0.28	0.36	0.42	0.27	0.41
16	KL	2	0.23	0.22	0.38	0.41	0.47	0.63
17	KG	3	0.14	0.26	0.25	0.44	0.45	0.68
18	MMRH	5	0.22	0.26	0.35	0.40	0.37	0.63
19	MRTH	4	0.33	0.20	0.48	0.31	0.37	0.74
20	NMMT	3	0.16	0.18	0.25	0.26	0.34	0.43
21	NIK	4	0.19	0.24	0.30	0.36	0.40	0.72
22	OR	3	0.22	0.20	0.35	0.30	0.30	0.42
23	PJ	2	0.26	0.27	0.42	0.46	0.45	0.50
24	RS	2	0.25	0.26	0.41	0.42	0.42	0.63
25	SK	6	0.44	0.49	0.62	0.79	0.54	0.90
26	SIK	3	0.19	0.20	0.28	0.34	0.40	0.55
27	SKK	1	0.17	0.16	0.28	0.27	0.28	0.46
28	TN	2	0.25	0.22	0.40	0.45	0.45	0.65
29	TL	3	0.23	0.28	0.37	0.41	0.33	0.54
30	UK	2	0.29	0.35	0.44	0.54	0.44	0.76
31	VDH	2	0.24	0.20	0.39	0.30	0.30	0.55
32	WMP	3	0.25	0.29	0.38	0.44	0.30	0.51
33	WRJ	6	0.37	0.40	0.65	0.55	0.52	0.90
34	WUP	2	0.34	0.27	0.51	0.41	0.30	0.55

∆Hq = spread of Generalized Hurst Exponent (GHE), ∆hq = width of the multifractal spectrum.

The Hurst exponent, also known as the Hurst index (HI: H (q = 2)) is well recognized as a metric for assessing predictability, particularly when its value exceeds 0.5. In locations characterized by greater Hurst exponent values, the occurrence of meteorological and agricultural droughts may be easily forecasted and anticipated^63^. Table 2 presents the results regarding the Hurst exponent values and their implications for all 34 meteorological sub-divisions. It was observed that most of the regions are showing short-term persistence (STP), in the SPI-n time series selected from the propagation analysis. Hurst exponent value generally increases with the SPI aggregation time scale as reported in one of the previous study^29^, which is also observed in current analysis’ results, as mostly the regions with the long-term persistence (LTP), have higher values of DPT [J & K: SPI-4 (HI = 0.75); SK: SPI-6 (HI = 0.54); WRJ: SPI-6 (HI = 0.52)]. However, for the SSMI-1 time series, almost ~ 38 % of the regions has shown the STP (HI < 0.5), whereas, ~ 62 % of the Indian regions have shown the LTP (HI > 0.5). The degree of persistence varied among the sub-divisions for SPI-n and SSMI-1 time series, with the lowest value for JKH (HI = 0.29); highest value for J & K (HI = 0.75) for SPI time series. For SSMI-1 time series, lowest HI is observed for CG (0.4) and highest for SK (0.91) region, with the Hurst exponent values for most of the humid regions is less than 0.5, showing the existence of STP, whereas, for most of the arid regions, it is more than 0.5, showing the LTP in the respective time series (see Table 2).

**Figure 6:**
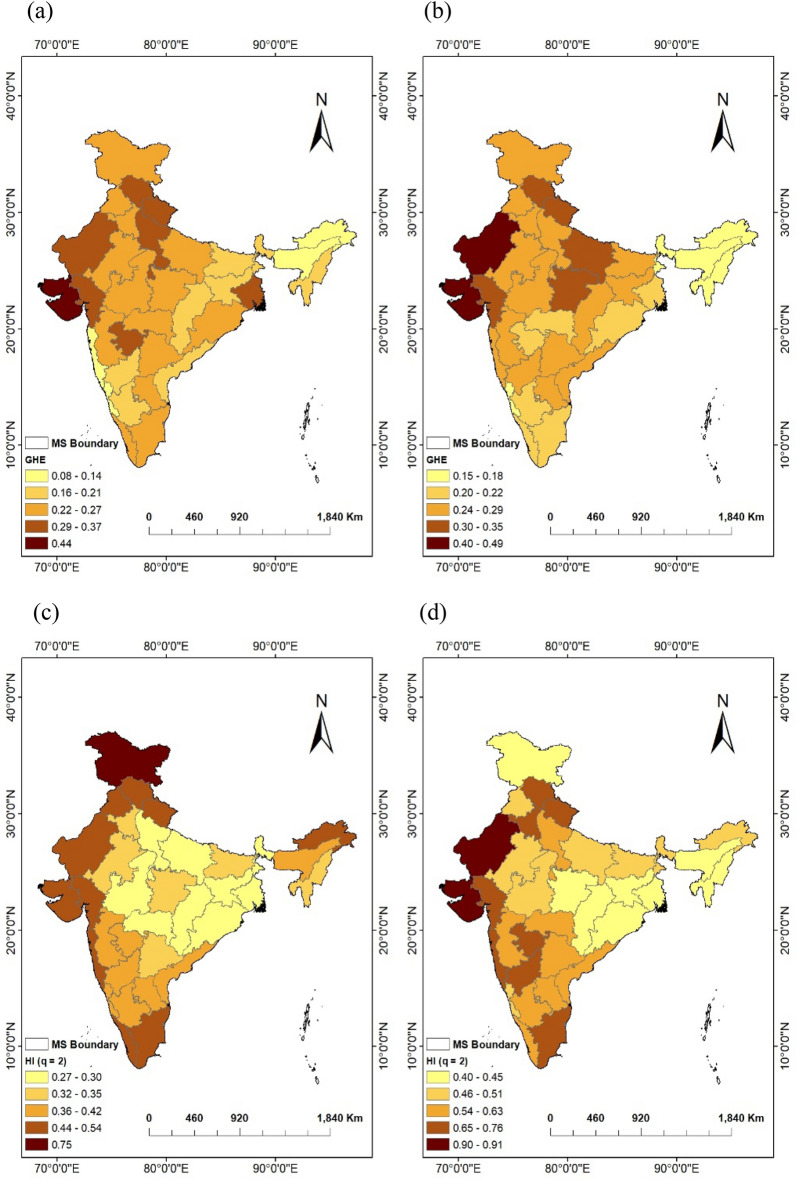
Spatial variation of (**a** and **b**) spread of Generalized Hurst Exponent (GHE: ∆Hq), (**c** and **d**) Hurst Exponent/Index at q = 2 (HI) for DPT-based SPI time series and SSMI-1 time series respectively, over the meteorological sub-divisions (MS) of India. (The Figure was generated by ArcGIS 10.3 software, https://www.esri.com/en-us/arcgis/products/arcgis-desktop/resources).

These findings suggest that areas characterized by arid and semi-arid climates tend to have longer propagation times from meteorological to agricultural drought and exhibit more robust long-term persistence in the SPI and SSMI time series. These results also revealed the sub-division where agricultural and meteorological droughts are better and easily predictable^64^, have strongly persistent time series with higher Hurst exponents. For all of India's sub-divisions, no clear relationship between DPT and Hurst exponent was found, however dry climate regions with higher DPT were found to have a more evident relationship as shown in Fig. 6c,d. Furthermore, the present study also reveals a more pronounced relationship between the HI value of the SSMI-1 time series and climate type of different regions.

The spatial variation of GHE values corresponding to a range of − 5 to 5, and the Hurst index at moment order q = 2 across meteorological subdivisions of India are represented in Fig. 6a–d for the meteorological and agricultural drought indicators (SPI-n,SSMI-1). In general, regions with shorter DPT exhibit lower values for the ∆Hq (spread of GHE) and H(2) (Hurst index at q = 2). Specifically, the regions of AP, CK, AM, and SKK show the lower DPT of 2 months, with corresponding ∆Hq values of 0.08, 0.11, 0.13, and 0.17 for SPI time series, and 0.16, 0.17, 0.13, and 0.16 for SSMI-1 time series. Conversely, arid and semi-arid regions exhibit higher DPT values, leading to increased multifractality in the associated SPI time series, which is evident from larger values of Hq. For instance, the GJ, WRJ, and SK regions with the highest DPT (6 months) show ∆Hq values of 0.35, 0.37, and 0.44 for SPI-6, and 0.31, 0.4, and 0.49 for SSMI-1 time series (see Fig. 6a,b).

**Figure 7:**
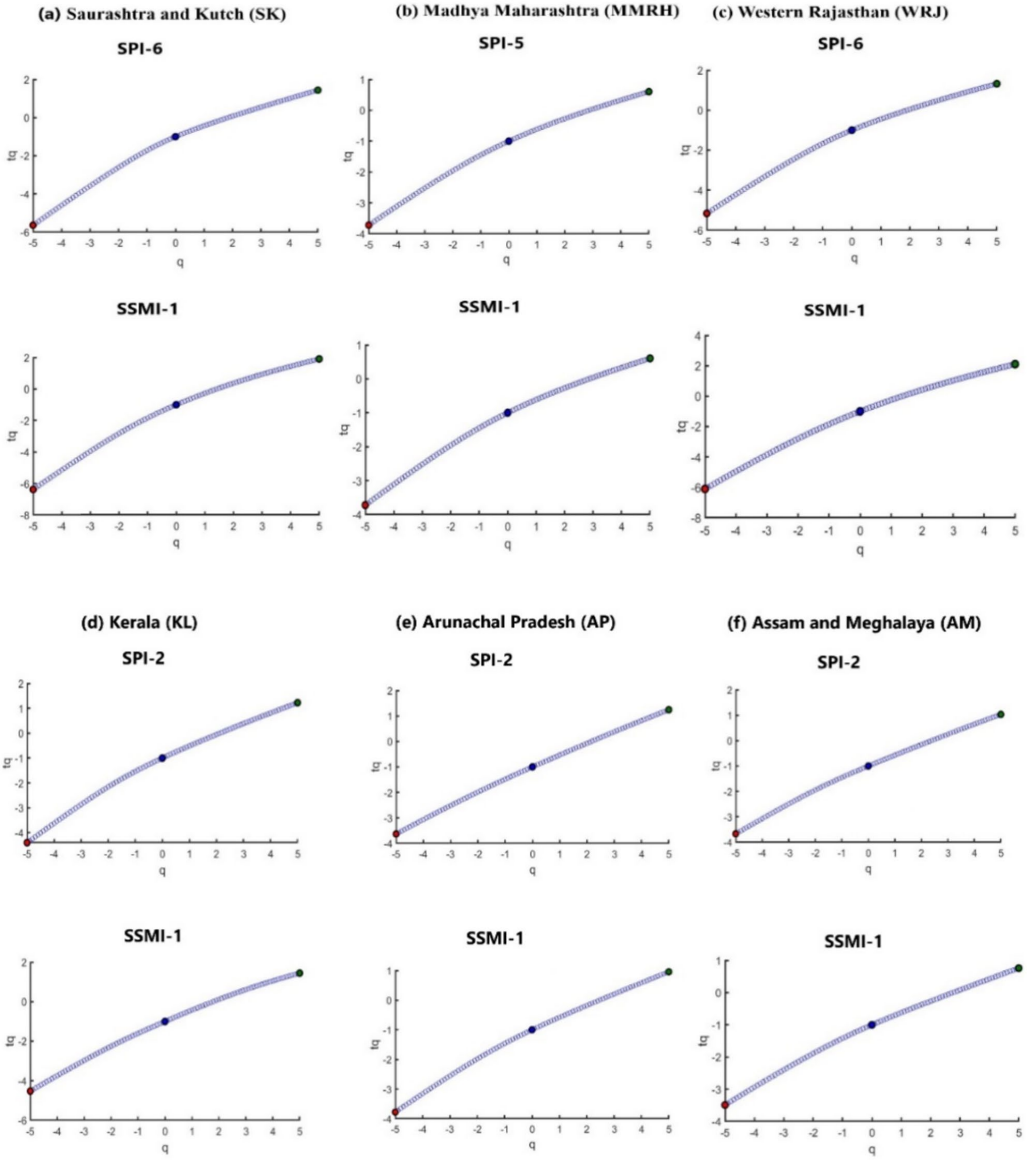
q- order mass exponent plots for DPT-based SPI time series and SSMI-1 time series showing the evidence of multifractality for six representative meteorological sub-divisions.

A comprehensive analysis of the multifractal nature of the time series can be achieved by examining the variation of the mass exponent for different moment orders ‘q’ (tq vs q). As depicted in Fig. 7 below, for the six selected representative regions, the mass exponent exhibits a curved dependence on moment order q, indicating the presence of multifractality in all regions with distinct climate types. Distinct slope values are observed in the tq vs q plot before and after the point q = 0, and the discrepancy in these slope values provides insights into the degree of multifractality. In the illustrated regions, the differences in slope for the SPI time series are 0.46 (SK), 0.23 (MMRH), 0.38 (WRJ), 0.24 (KL), 0.07 (AP), and 0.13 (AM). Correspondingly, for the SSMI time series, these differences are 0.27 (SK), 0.47 (MMRH), 0.41 (WRJ), 0.22 (KL), 0.17 (AP), and 0.15 (AM). These results indicate that arid climatic regions (SK, MMRH, and WRJ) exhibit higher values for slope differences, signifying a higher degree of complexity and multifractality compared to humid regions (KL, AP, and AM). The convex nature of all curves in Fig. 7 demonstrates that all regions deviate significantly from the monofractal case and possess multifractal characteristics with varying degrees of complexity. According to the findings, dry locations with higher DPT exhibit characteristics such as higher multifractal degree and complexity. Similarly, regions with lower DPT in humid climates are associated with lower degrees of multifractality in both SPI and SSMI time series.

**Figure 8:**
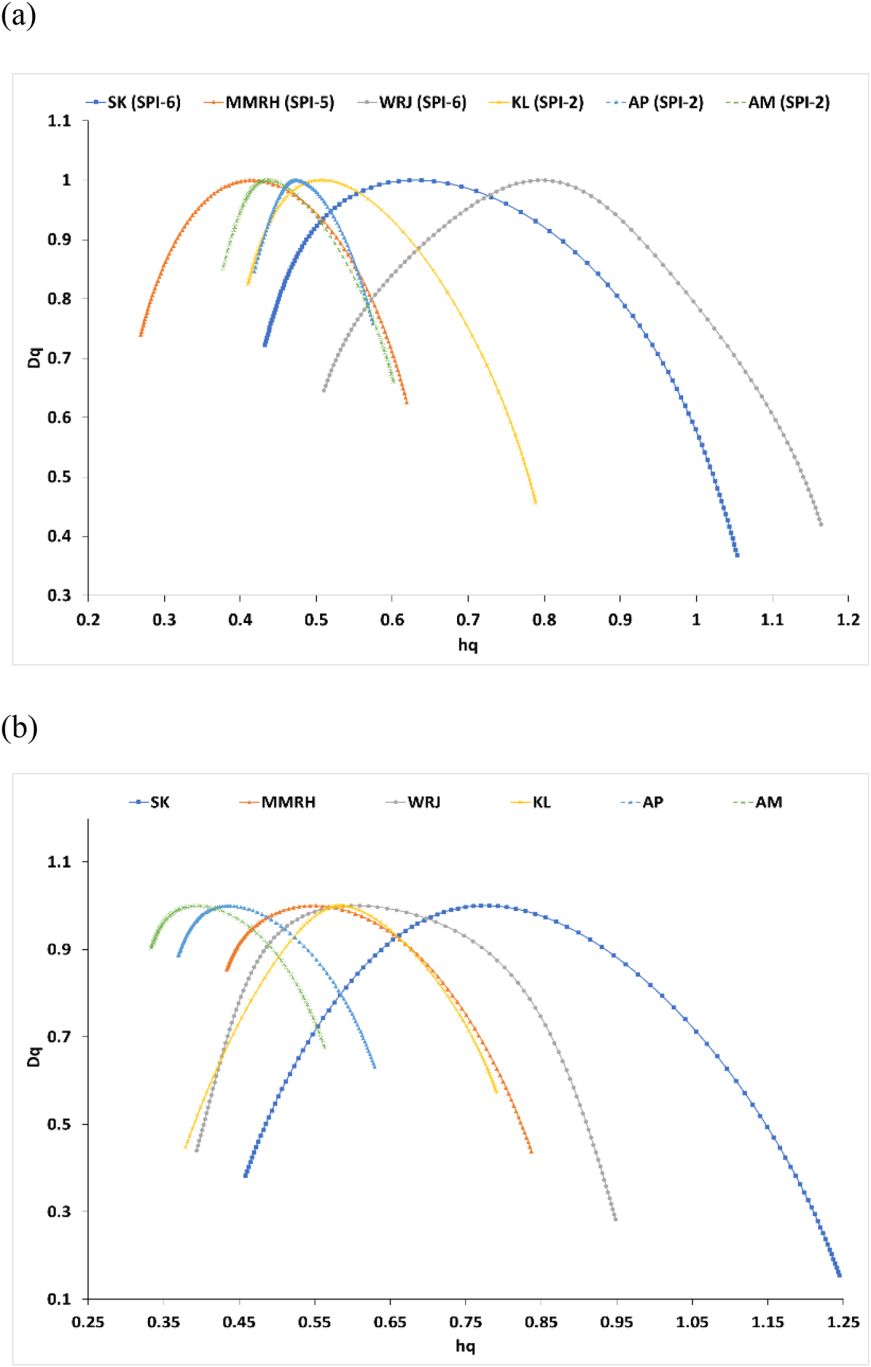
Multifractal spectra of (a) DPT based SPI time series and (b) SSMI-1 time series for 6 representative meteorological sub divisions.

The multifractal spectrum of the SPI and SSMI time series for the selected regions was also studied for the detailed investigation into the degree of multifractality as depicted in Fig. 8a,b. The asymmetric, inverted shape parabola is representative of the multifractal degree in the time series. Left and right limbs of the spectra correspond to the spread of high or low fluctuations in the time series for the selected meteorological sub-divisions as shown in Fig. 8a (for SPI) and Fig. 8b (for SSMI)^57,65^. The presence of multifractality is strongly supported by the observation of asymmetric inverted parabola curves, which confirms the existence of dissimilarity in scaling behavior for different climatic subdivisions, especially between humid and arid regions as mentioned in the previous sections. Also, it is evident that the multifractal spectrum’s width (∆hq) expands as the DPT in terms of aggregation timescale increases (see Table 2), showing the clear linkage between degree of multifractality and DPT. Significant variation across sub divisions with different climatic conditions and DPT was inevitably shown by the multifractal features. From Table 2, it can be seen that, for the WRJ region having the highest DPT (6—months), multifractal parameters for the SPI time series (∆Hq = 0.37, ∆hq = 0.65, HI = 0.52) and SSMI time series (∆Hq = 0.40, ∆hq = 0.55, HI = 0.90) are on the higher side. Whereas, regions with humid climatic conditions (KL, AM, and AP) have lower values for corresponding multifractal parameters. Thus, it implies that locations with more dry or semi-arid climates are showing the dominant presence of multifractal degrees than those with humid climates.

**Figure 9:**
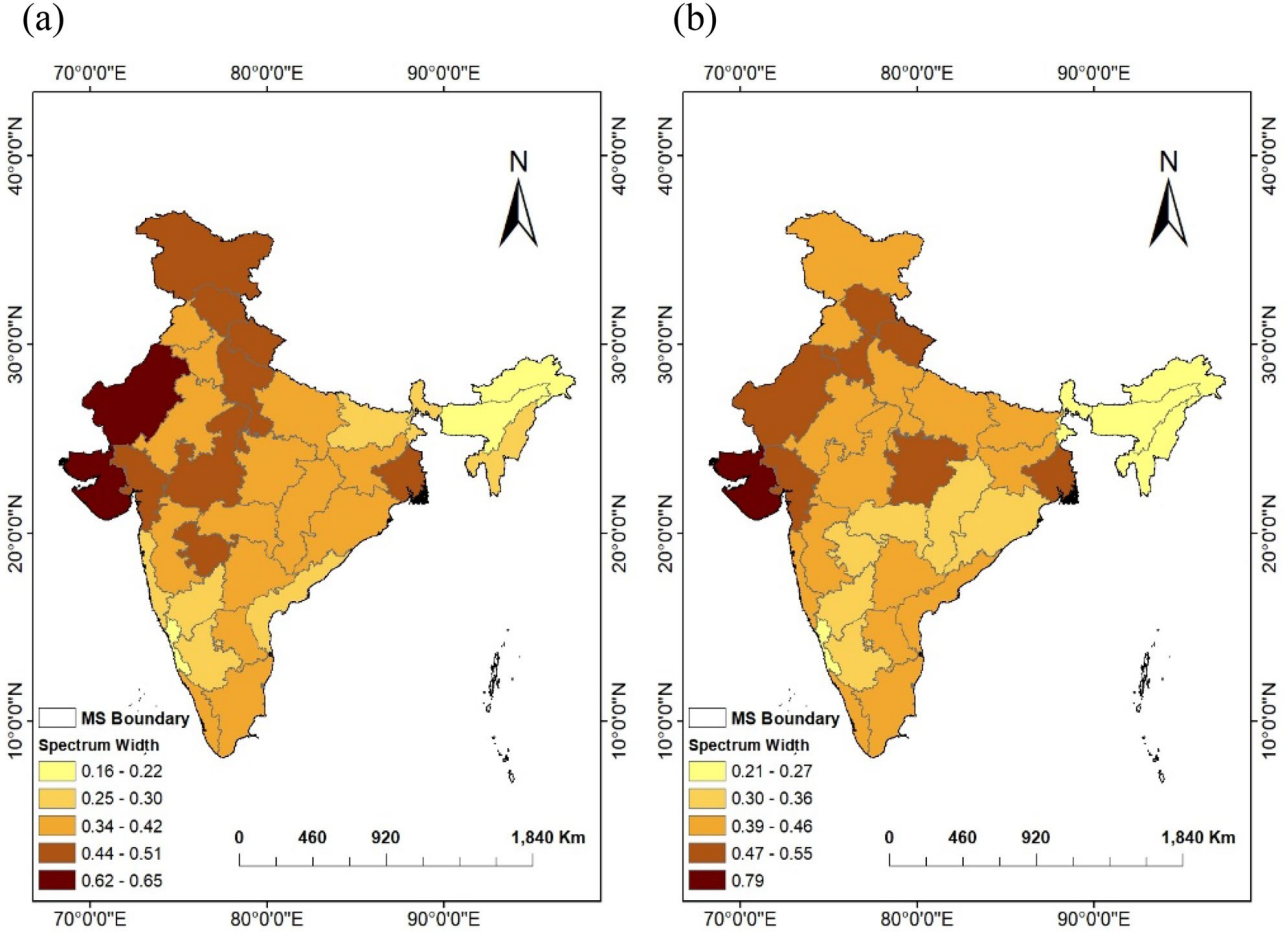
Spatial variation of the width of multifractal spectrum (∆hq) for (a) DPT-based SPI time series and (b) SSMI-1 time series over meteorological subdivisions (MS) of India. (The Figure was generated by ArcGIS 10.3 software, https://www.esri.com/en-us/arcgis/products/arcgis-desktop/resources).

The spatial variation of the multifractal spectra width for all meteorological subdivisions of India is shown in Fig. 9. Multifractal degree in terms of the width of multifractal spectra (∆hq) varies from 0.16 (AP: DPT of 2 months) to 0.65 (WRJ: DPT of 6 months) for SPI time series and 0.21 (CK: DPT of 2 months) to 0.79 (SK: DPT of 6 months) for SSMI time series. WRJ and SK regions having the highest DPT (6 months) have also shown higher values for the width of multifractal spectra as 0.65 and 0.55; 0.62 and 0.79 for the SPI and SSMI time series respectively, showing evidence for a higher degree of multifractality. From the result of the present study, it was found that, for meteorological sub-divisions, multifractal spectra are inverse asymmetric parabola, confirming the presence of multifractality with no definite link with DPT but in general having higher values for arid climatic regions.

## Discussion and conclusions

The primary goal of this study was to investigate the scaling patterns of meteorological and agricultural drought propagation properties quantified using appropriate drought indices and to apply multifractal analysis in India’s meteorological sub-divisions to see its dissection between humid and arid land areas. The MF-DFA application in SPI and SSMI drought indices offers a comprehensive and advanced approach for analyzing the multifractal properties and complex dynamics of drought patterns. Multifractal characterization provides useful information on the long and short-term memory in the drought index time series, which can yield the information on the future trend of drought events in the region^29,66^. Such an analysis provides valuable insights into drought persistence, its scaling behavior, and multifractal characteristics of drought indices, leading to a deeper understanding of the evolution of drought phenomena and improved drought prediction for efficient drought management. Many authors in the past have studied the multifractality^29,55,56^ and propagation in the drought time series separately without bothering about the inherent links between these processes. The current work aims to investigate the unexplored links of multifractal-based characterization with drought propagation mechanisms. Studying the multifractality and propagation together is important for a comprehensive understanding of drought dynamics and their implications^8,66^. Most of the recent works on drought propagation in the context of agricultural drought is confined to studying the response time, and there is a less focused analysis of the associated multifractality/persistence in the long-term data of the meteorological/soil moisture variables. Combining the multifractal-based analysis with the drought propagation is more significant as compared to the traditional studies, as, in this approach, we are studying the SPI time scale which is more responsible for the soil moisture-based agricultural drought, and scaling behavior and persistence/memory information in the selected SPI time scale is more significant from the agricultural drought point of view^27,29,66^. Contrary to general expectations, our study results suggest that humid regions exhibit lower linear DPT and lower levels of multifractality in drought index time series than their arid counterparts of India. These study results are in line with the findings reported in past study^6^ indicating that less DPT for China’s humid regions (3 months) than the arid and semi-arid areas (up to 8 months). In the present study, drought propagation from meteorological (SPI-n) to agricultural drought (SSMI-1) was analyzed using the linear approach of MPCC, and the DPT is estimated based on SPI timescale showing the highest correlation with SSMI-1. Though this approach is yielding meaningful results, some studies are highlighting the need to establish both linear and nonlinear connections among different drought types to completely understand the complex drought propagation mechanism^7,67,68^.

DPT is highly variable over meteorological sub-divisions of India following the climatic gradient of the region; though the uniform pattern is not observed everywhere, as propagation dynamics could also affected by many additional factors such as, human influence in terms of supplementary irrigation, groundwater resources, etc and should be addressed in future researches. A similar study has been conducted by few researchers^22,69^ and reported that regions with different climatic settings produce variable drought propagation mechanisms. Results from the present study have confirmed these findings, showing that regions with arid/semi-arid climate like GJ (5.67 months), SK (5.64 months), and WRJ (5.73 months) has shown higher values of annual average DPT (see Table 1), additionally, the investigation of the associated multifractality with the DPT is done which is lacking in the previous studies. Whereas, regions with humid and wet climate having the dominance of south west and north east monsoon has shown lower values of annual average DPT [AM (2.00 month), AP (1.75 months), KL (2.29 months), and SKK (1.20 months)] as soil moisture is responding at the shorter timescale of SPI. The variation and synchrony of the DPT with the long-term climatic condition/aridity of the region and across the seasons are observed as soil moisture depletion is largely affected by precipitation and evaporative demand^10,16^ which is highly variable (both spatially and temporally) in the tropical monsoon climate of India^70^. Meteorological drought occurring because of the anomalous precipitation can directly affect soil moisture depletion and surface water (streamflow), whereas, reduced soil moisture can negatively impact the runoff generation process, similarly, reduced streamflow can propagate to the long-term agricultural droughts. The drought propagation process is interconnected and the interplay between different water spheres (precipitation, soil-water, and streamflow) is complex, as the meteorological drought is triggering the other drought types (agricultural and hydrological droughts) at varying lag times^8,10^. Higher DPT for arid climatic regions may be because these regions have the presence of dry soil and whatever scanty rainfall occurs in these areas may not immediately reflect in the increase in RZSM, hence showing the weak linkage between rainfall and RZSM. Similar results were reported in previous works done^6,71^, mentioning that arid climatic regions show weak linkages between precipitation and soil moisture as the other climatic elements may also be affecting the soil water stress in these regions such as soil type, wind, radiation, etc. A shorter propagation time for humid areas is observed which can be linked with higher evapotranspiration connected with the warming weather resulting in quick alterations of soil moisture to rainfall anomaly. These observations are in line with the findings reported in the past studies^62,72^, which highlight the lower propagation time for humid regions.

The present study has reported that the multifractality, scaling behavior, and persistence (see Table 2) in the SPI time series important from the agricultural drought perspective with varying aggregation time scales along with the SSMI-1 time series shows significant spatial variation for all meteorological sub-divisions. Similar variability has been reported in past studies^29,66^, which can be attributed to the climatic aridity, soil type, and regional availability of the supplementary irrigation facilities^8^. Results from the current analysis of drought indices (SPI and SSMI) revealed a noteworthy relationship between the Hurst exponent and the predictability of drought in different regions of India as it is an indicator of persistence and long-term memory in time series data^29,63,73^. Our study revealed higher predictability and therefore enhanced forecast ability in regions like HP, WRJ, and SK due to higher Hurst exponent in SPI time series; the same LTP is observed for the ~ 62 % of the meteorological regions (mostly arid and semi-arid) in the SSMI-1 time series (see Table 2) and this information can be attributed to the underlying temporal structure, indicating a certain level of consistency and regularity in the occurrence of drought events. From the observed LTP in the arid regions, it can be inferred that the trend of agricultural droughts and soil moisture drying events will continue in the future as well. A similar observation is reported in the study done over the California and Mississippi watersheds^66^ showing LTP for the SPI time series studied. In the current study, for the SPI time series, most of the regions have shown the STP (HI < 0.5) for the selected time scale based on the DPT (see Table 2). The findings of the STP analysis on the SPI time series, specifically at a time scale of DPT, align with the results in the past literature^29^. Their study reveals that, Hurst exponent values exhibit an upward trend as the aggregation time scale increases while displaying lower values for lower SPI time scales. STP observed in the SPI and SSMI time series is important for modeling the short-term droughts in the respective regions. In the past studies^29,65^ it is reported that the variation of multifractal properties in SPI and SPEI time series respectively with different aggregation timescales highlighting the increase in degree of multifractality in terms of the width of the multifractal spectrum (∆hq) with increasing spread of the GHE (∆Hq) and aggregation timescales of drought index. Similar evidence has been reported in the present study, as the regions with higher DPT (higher SPI timescales) were also showing a higher degree of multifractality, e.g., DPT for SK, MMRH, and WRJ is 5.64, 5.00, and 5.73 months respectively and degree of multifractality in terms of the width of multifractal spectrum for the same regions are 0.62, 0.35, and 0.65 in SPI time series, whereas, it is 0.79, 0.40, and 0.55 in SSMI time series. A similar close link between the degree of multifractality and DPT was observed for humid climatic regions like AM, KL, and AP showing the presence of a lower degree of multifractality and lesser DPT (see Table 2).

As a word of caution, it is important to note the limitations of our study in terms of datasets and limited ground variables used. The study solely focused on meteorological and soil moisture datasets, neglecting local agricultural practices and existing surface conditions (i.e. vegetation type, irrigation) and their influence on drought dynamics. For agrarian countries like India, with the considerable presence of supplementary irrigation facilities and spatially varying land use, and soil type characteristics, meteorological drought may not always lead to agricultural drought due to weak correlation (see Fig. S3 of the supplementary material), but can indirectly set the trigger by affecting the overall catchment water storage of surface water and groundwater^8,74^. A recent study^75^ has demonstrated that areas with intensive agriculture and irrigation, featuring multiple cropping seasons per year, experience significantly shorter drought propagation times compared to regions with single cropping or no irrigation. Further, to highlight the role of human management practices such as irrigation and reservoir operation on drought and water budget components, a synthesis of past studies is demonstrated in Table S2 of the supplementary material. Hence, future research could explore the underlying multifractal nature of how various vegetation and irrigation types influence drought propagation times across different climate zones in India. If we consider the impact of irrigation on drought propagation, agricultural drought doesn't solely correlate with meteorological drought (precipitation deficiency) especially in regions and river basins where water regime is highly influenced by human activities. This disturbed natural connection of meteorological drought with soil moisture and vegetation based agricultural drought can be observed through the heatmaps showing the average annual values of SPI-1, SSMI-1, and sNDVI (standardized anomaly of NDVI) from 1982 till 2020 (overlapping period) (see section “Spatio-temporal variation of SPI-1, SSMI-1, and sNDVI” of the supplementary material). It can also relate to hydrological drought (deficiency in surface and groundwater), along with other anthropogenic factors like reservoir operations and land management activities^8,10^. The present study utilizes the ERA5 soil moisture data, which is based on a combination of model simulation and data assimilation^40^. By virtue of the latter, irrigation estimates can be partially reflected in assimilated soil moisture simulations^76^ leading to possibly a weaker correlation between meteorological and soil moisture drought signals. Notably crop growth activities with rainfed and irrigation modules need to be accounted for a full-length consideration of extra water content as irrigation that is supplemented to the root-zone soil moisture other than rainfall. Further, for a comprehensive understanding, localized analyses at a catchment or regional scale, it is recommended the incorporation of hydrological drought (surface and groundwater deficiency) data to study the interconnection between all three drought types which could improve the inference on the drought propagation time results^10^.

The similar analysis has been extended to the vegetation-based drought index i.e., sNDVI for the assessment of the agricultural drought and then underlying multifractal nature is explored for different climatic regions (see section “Multifractal features of sNDVI time series” of the supplementary material). The similar inference is observed from the multifractal assessment of the sNDVI time series as that for SPI and SSMI-1, showing the higher strength of multifractality for the drier regions and lower values for the wet climatic regions (see Table S1, Figs. S4 and S5 of the supplementary material). This makes it imperative to trust the soil moisture-based multifractal assessment, as it proven to have similar multifractal dynamics as that of observed for the vegetation health-based measure (sNDVI). Overall, this study advocates that identifying regions with distinct DPT and knowing the multifractal nature of highly correlating SPI and SSMI timescales. The combined assessment of the drought propagation and multifractality/persistence is more significant ^66^, compared to the traditional studies, as drought management and mitigation efforts can be focused more effectively, allowing for better resource allocation and preparedness strategies in regions with a higher likelihood of experiencing persistent and recurring drought conditions.

The main findings from this study are:

- The study establishes a unique linkage between drought propagation mechanism and multifractality, which is influenced by the climate of the meteorological regions in India.
- Regions with higher DPT and arid/semi-arid climates exhibit greater multifractality and long-term persistence in drought patterns compared to humid regions in India.
- Multifractality is evident in all DPT-based SPI time series across different regions, with notable non-linear dependence of Generalized Hurst Exponent (GHE) and mass exponent for moment order q, and an inverted asymmetric nature of the multifractal spectra.
- The Hurst index values of SPI time series in most meteorological subdivisions are below 0.5, indicating the short-term persistence of drought, observed mostly because of the lower SPI time scales^29^ which is likely to continue in the future. Whereas, HI for SSMI time series, shows mixed response of STP (38% of the meteorological regions) and LTP (62% of the meteorological regions).
- Specific example demonstrates the influence of DPT on multifractality: the Western Rajasthan and Saurashtra and Kutch region, with the highest annual average DPT of 5.73 and 5.64 months (SPI time scale of 6 months), shows the value of 0.65 and 0.62 for the width of multifractal spectra. In contrast, for the Sikkim region, with the lowest DPT of 1.20 months (SPI time scale of 1 months), it is 0.28.
- The multifractal degree rises with an increase in DPT, being higher in arid regions and lower in humid regions, providing valuable insights into drought forecasting studies for different meteorological subdivisions in India.

### Supplementary Information

Supplementary Information.

## Data Availability

Data used for the present work are available online: precipitation data from India Meteorological Department (https://www.imdpune.gov.in/lrfindex.php), soil moisture data from Copernicus Climate Data Store (https://cds.climate.copernicus.eu/cdsapp#!/dataset/reanalysis-era5-single-levels?tab=form), NDVI data from NASA/GFSC Global Inventory Modelling and Mapping Studies (GIMMS) (https://www.ncei.noaa.gov/metadata/geoportal/rest/metadata/item/gov.noaa.ncdc:C01558/html), and Terra Moderate Resolution Imaging Spectroradiometer (MODIS) Vegetation Indices 16-Day (MOD13A1) Version 6.1 product (https://lpdaac.usgs.gov/products/mod13a1v061/). The results/data generated from the study can be made available from the corresponding author on reasonable request.
