# Supplementary material for: Multifractal characterization of meteorological to agricultural drought propagation over India
Source: Sci Rep. 2024 Aug 14;14:18889. doi: 10.1038/s41598-024-68534-0 (PMC11324948; doi:10.1038/s41598-024-68534-0)
Supplement: Supplementary file 1 — Supplementary Information. [file 41598_2024_68534_MOESM1_ESM.docx]

**Supplementary Material**

**Multifractal characterization of meteorological to agricultural drought propagation over India**

Akshay Bajirao Pachore^1^, Renji Remesan^1*^, Rohini Kumar ^2^

^1^School of Water Resources, Indian Institute of Technology Kharagpur, India.

[*renji.remesan@swr.iitkgp.ac.in](mailto:*renji.remesan@swr.iitkgp.ac.in)

^2^ Helmholtz Centre for Environmental Research — UFZ, Leipzig, Germany.

1. **Data (precipitation and soil moisture) pre-processing**

Figure S1 shows the workflow for pre-processing the precipitation and soil moisture data for the computation of the meteorological (SPI) and agricultural (SSMI) drought index. Precipitation from IMD is available at 0.25^0^ x 0.25^0^ spatial resolution and daily time step, which is first aggregated to the monthly time step by taking the sum of corresponding daily values. Soil moisture data is utilized from the ERA5 which is available at multiple depths, which are layer 1 (0-7 cm), layer 2 (7-28 cm), layer 3 (28-100 cm), and layer 4 (100-289 cm). For Indian conditions, the crop root zone depth selected for the present analysis is 60 cm, and hence layers 1, 2, and partly 3 are aggregated to get the soil moisture data of the top 60 cm depth. The third layer is partially integrated – assuming the uniformity of soil moisture over the depth of 28 cm to 60 cm. Regarding the temporal information, ERA5 is available at the hourly time step, which is averaged to the monthly time step by taking an average of hourly values to get the daily estimates and further taking an average of daily values to get the monthly soil moisture estimates. Equation 1 (Zha et al., 2023) is used to get the temporal and depth-wise aggregation of the soil moisture data.

RZSM = $\frac{1}{24}\sum_{t=1}^{24} \sum_{i=1}^{3} (D_{i} \times\theta_{i,t})$ (1)

Here, RZSM is the root zone soil moisture, soil layer depth is denoted by D_i_ (D_1_ = 7 cm, D_2_ = 21 cm, D_3_ = 32 cm), and $\theta_{i,t}$ is the volumetric soil water content (m^3^/m^3^).

After aggregating the precipitation and soil moisture data to the monthly scale, the spatial average is taken for each of the 34 meteorological sub-divisions of India. Further, the drought index computation, drought propagation time estimation, and multifractal analysis are done for all meteorological sub-divisions independently to analyze the spatial variation as per the different hydrometeorological conditions of these sub-divisions.


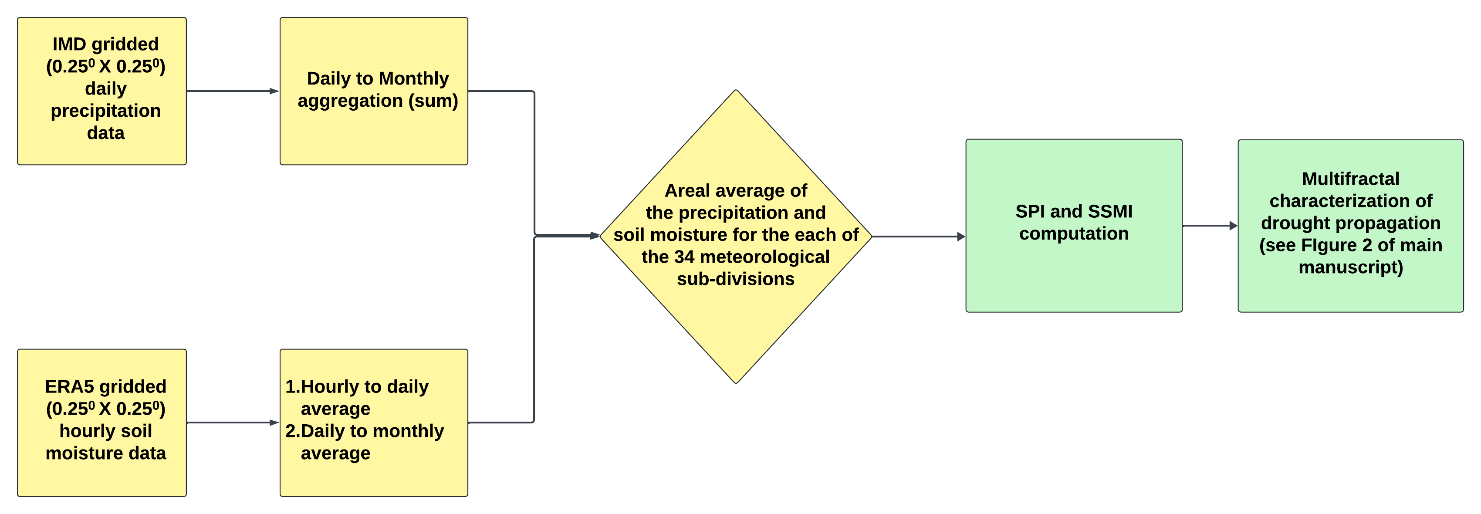


Figure S1: Flow chart depicting the pre-processing of the input data for drought index (SPI and SSMI) computation and multifractal characterization using MF-DFA.

1. **Spatio-temporal variation of SPI-1, SSMI-1, and sNDVI**

To depict the connection between monthly rainfall anomaly (SPI-1), soil moisture anomaly (SSMI-1), and NDVI anomaly (sNDVI) heatmaps showing the average annual values of these indices is plotted (see Figure S2) for the overlapping period from 1982 till 2020. The NDVI values are converted to the standardized anomaly values to make comparison for valid among indices, as other two (SPI and SSMI) are also the standardized anomalies of rainfall and soil moisture respectively. Standardized anomaly of NDVI is calculated using the same equation used for SPI computation (equation 1 of the main manuscript) and uses the monthly averaged NDVI values instead of precipitation and represented by sNDVI.

1. **SPI-1**


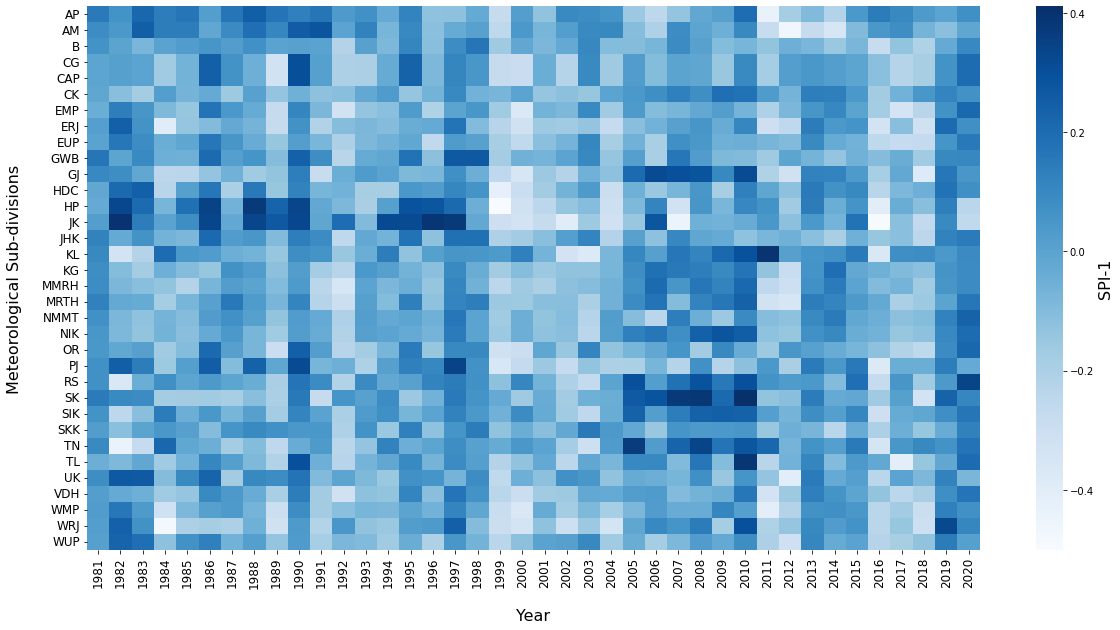


1. **SSMI-1**


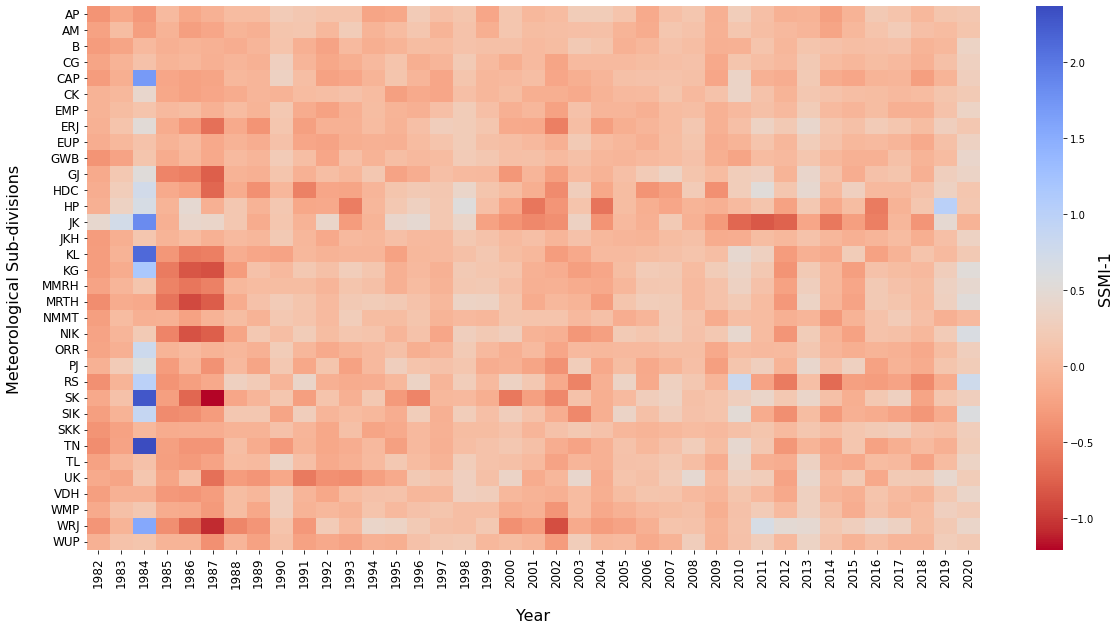


**(c) sNDVI**

**
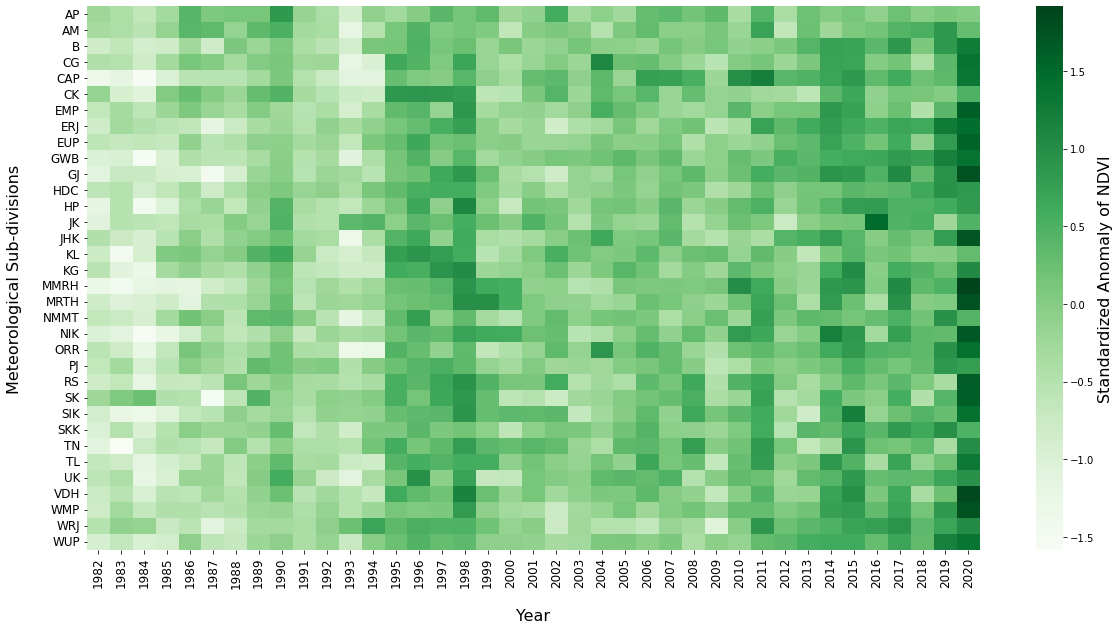
**

Figure S2: Annual average (a) SPI-1, (b) SSMI-1, and (c) sNDVI (standardized anomaly of NDVI) for all 34 meteorological sub-divisions from 1982 till 2020.

1. **Average annual PCC of SPIn-SSMI-1 and SPIn-sNDVI**

The Pearson’s correlation coefficient (PCC) values are computed for each month, between SPIn (n varies from 1-month, 2-month,…., 12-months) and SSMI-1, sNDVI. Further the annual averages are calculated over 12 months and represented in the form of heatmaps (see Figure S3). The weak annual average correlations are observed for SPIn-SSMI-1, however, this becomes weaker for SPIn-sNDVI and can be seen from the correlation heatmaps (see Figure S3).

**(a)**


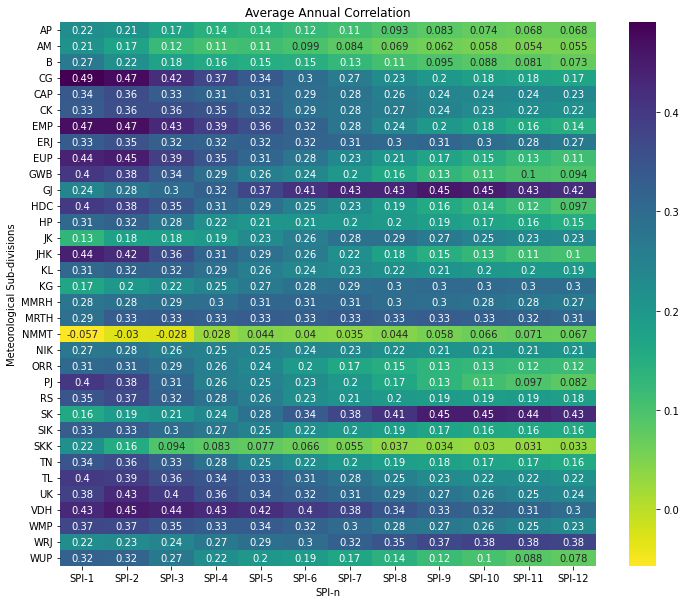


**(b)**

**
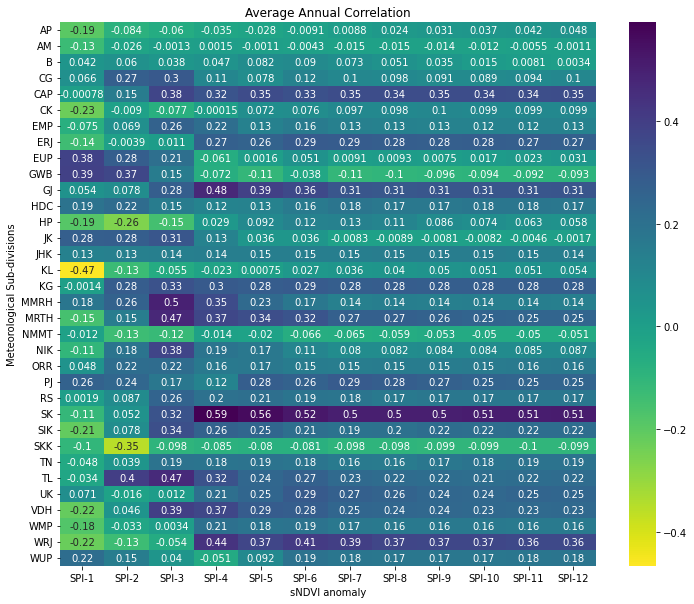
**

Figure S3: Average annual PCC for (a) SPIn – SSMI-1 and (b) SPIn- sNDVI for time series data from 1982 till 2020 for all 34 meteorological sub-divisions.

1. **Multifractal features of sNDVI time series**

**
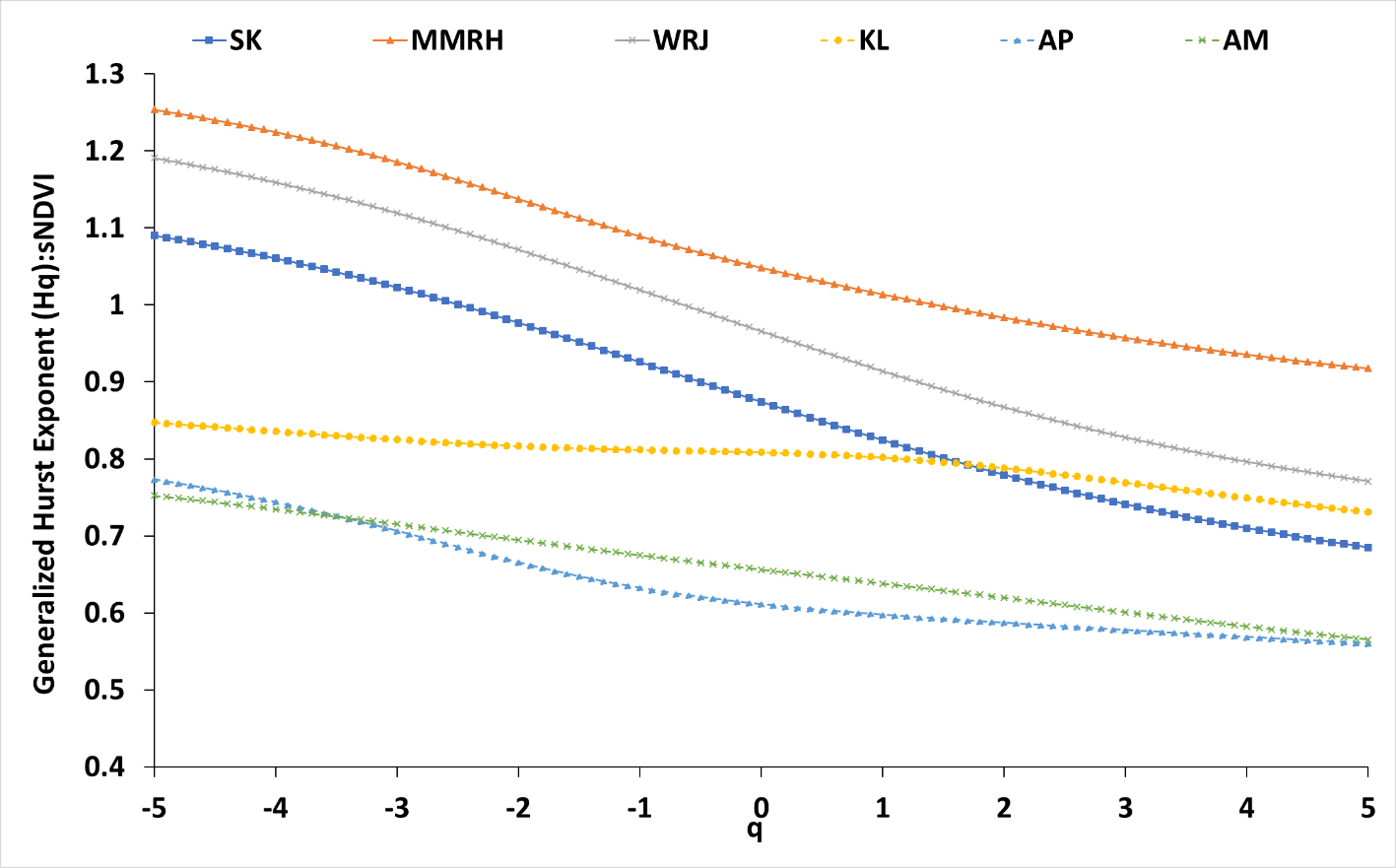
**

Figure S4: Generalized Hurst Exponent (GHE) plots for sNDVI timeseries for six representative meteorological sub-divisions.

**
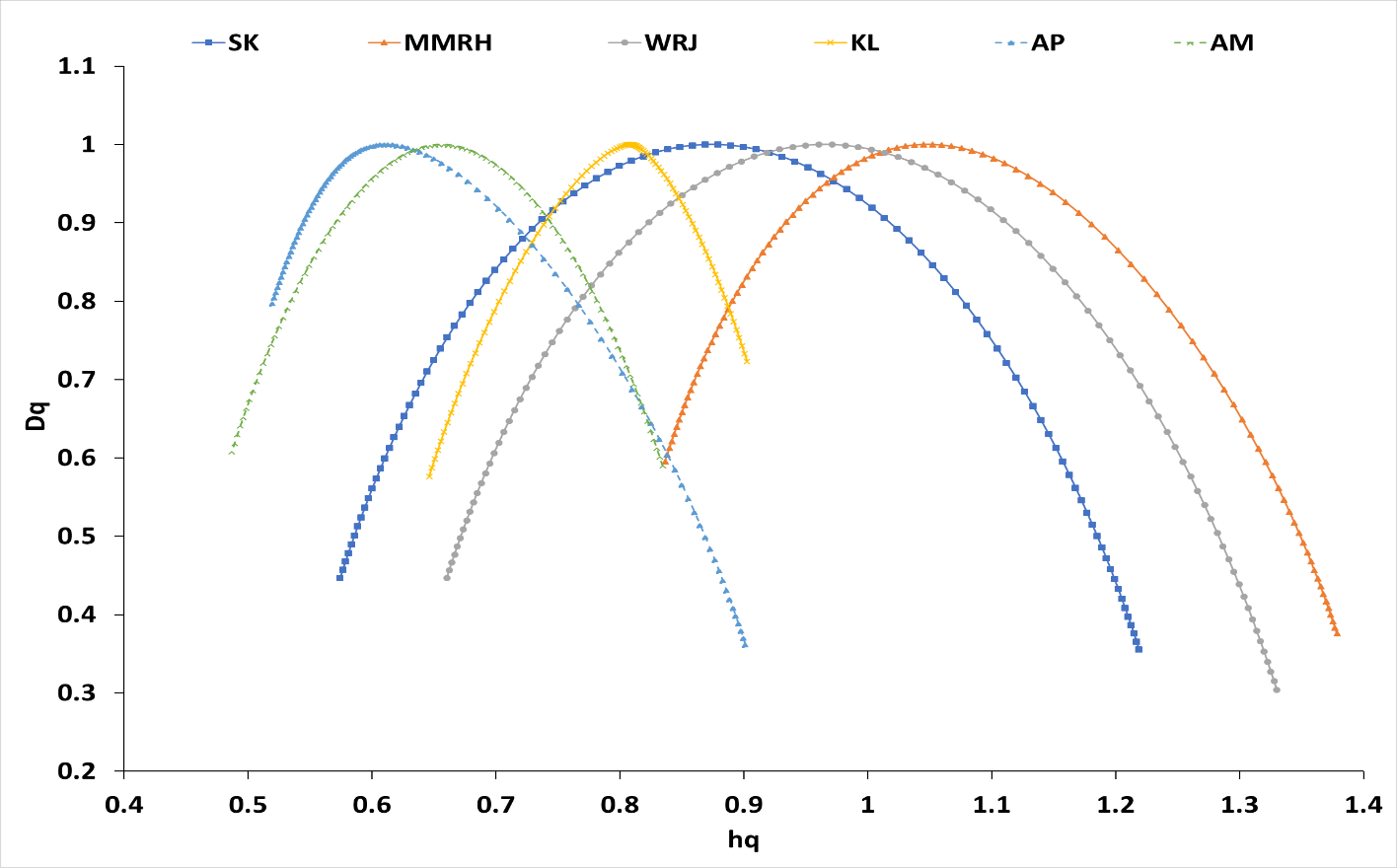
**

Figure S5: Multifractal spectra of sNDVI time series for 6 representative meteorological sub divisions.

Table S1: Multifractal parameters for 34 meteorological sub-divisions of India for sNDVI time series.

| **Sr. No** | **Region** | **GHE range** **(∆Hq)** | **Spectrum width** **(∆hq)** | **Hurst Index (q =2)** |
| --- | --- | --- | --- | --- |
| 1 | AP | 0.21 | 0.38 | 0.59 |
| 2 | AM | 0.19 | 0.35 | 0.62 |
| 3 | B | 0.26 | 0.47 | 0.74 |
| 4 | CG | 0.37 | 0.62 | 0.71 |
| 5 | CAP | 0.27 | 0.43 | 0.94 |
| 6 | CK | 0.24 | 0.43 | 0.75 |
| 7 | EMP | 0.37 | 0.60 | 0.67 |
| 8 | ERJ | 0.29 | 0.51 | 0.85 |
| 9 | EUP | 0.34 | 0.57 | 0.70 |
| 10 | GWB | 0.26 | 0.45 | 0.88 |
| 11 | GJ | 0.27 | 0.46 | 0.94 |
| 12 | HDC | 0.25 | 0.42 | 0.74 |
| 13 | HP | 0.17 | 0.34 | 0.83 |
| 14 | J and K | 0.26 | 0.44 | 0.72 |
| 15 | JKH | 0.26 | 0.44 | 0.76 |
| 16 | KL | 0.12 | 0.26 | 0.79 |
| 17 | KG | 0.21 | 0.37 | 0.84 |
| 18 | MMRH | 0.34 | 0.54 | 0.98 |
| 19 | MRTH | 0.38 | 0.61 | 0.87 |
| 20 | NMMT | 0.19 | 0.35 | 0.70 |
| 21 | NIK | 0.32 | 0.55 | 0.94 |
| 22 | OR | 0.27 | 0.45 | 0.81 |
| 23 | PJ | 0.29 | 0.47 | 0.65 |
| 24 | RS | 0.25 | 0.44 | 0.83 |
| 25 | SK | 0.41 | 0.64 | 0.78 |
| 26 | SIK | 0.31 | 0.51 | 0.88 |
| 27 | SKK | 0.24 | 0.42 | 0.71 |
| 28 | TN | 0.24 | 0.51 | 0.83 |
| 29 | TL | 0.30 | 0.43 | 0.82 |
| 30 | UK | 0.23 | 0.41 | 0.79 |
| 31 | VDH | 0.43 | 0.71 | 0.78 |
| 32 | WMP | 0.34 | 0.58 | 0.73 |
| 33 | WRJ | 0.42 | 0.67 | 0.87 |
| 34 | WUP | 0.27 | 0.45 | 0.77 |

Table S2. Synthesis of the studies mentioning irrigation information in the context of drought analysis over India

| Sl. No | Research Paper | Major Highlights |
| --- | --- | --- |
|  | **Title:** Contrasting influence of human activities on agricultural and hydrological droughts in India  **Authors:** Shah, D., Shah, H. L., Dave, H. M., & Mishra, V  **Journal:** Science of the Total Environment  **Year:** 2021 | - Used the Community Water Model (CWatM) to analyse the impact of anthropogenic activities (irrigation, ground water pumping, and reservoir operation) on agricultural and hydrological drought in India. - Observed the increase in soil moisture and decrease in ground water storage under the human influence as compared to the natural/undisturbed scenario. |
|  | **Title:** Drought detection and declaration in India  **Authors:** Bhardwaj, K., & Mishra, V.  **Journal:** Water Security  **Year:** 2021 | - Analyzed the disparity between detected and declared droughts over all Indian districts. - High difference is observed in the irrigated regions for the declared and detected droughts. As the droughts get detected by monitoring framework but due to the presence of supplementary irrigation crop yield was maintained and drought was not declared. |
|  | **Title:** Strong Influence of Irrigation on Water Budget and Land Surface Temperature in Indian Subcontinental River Basins  **Authors:** Shah, H. L., Zhou, T., Huang, M., & Mishra, V  **Journal:** Journal of Geophysical Research  **Year:** 2019 | - Variable Infiltration Capacity model with irrigation scheme is used to analyze the influence of irrigation on water budget and land surface temperature. - Highlighted that irrigation increases the evapotranspiration in 18 river basins of India and alters the water budget of the region. |
|  | **Title:** Roles of irrigation and reservoir operations in modulating terrestrial water and energy budgets in the Indian Subcontinental River basins  **Authors:** Shah, H. L., Zhou, T., Sun, N., Huang, M., & Mishra, V.  **Journal:** Journal of Geophysical Research: Atmospheres  **Year:** 2019 | - Impact of irrigation and reservoir operation on terrestrial water budget of Indian river basins in examined. - Land surface hydrological model with consideration of irrigation scheme is coupled with water management model for the purpose of study. - Considerable impacts of irrigation and other management practices is observed on the water budget components during pre-monsoon season. |
|  | **Title:** Study on inter-seasonal and intra-seasonal relationships of meteorological and agricultural drought indices in the Rajasthan State of India  **Authors:** Dhakar, R., Sehgal, V. K., & Pradhan, S.  **Journal:** Journal of arid environments  **Year:** 2013 | - Crop response to meteorological drought is assessed by analyzing the relationship between SPI and VCI. - Reported that strength of connection between SPI and VCI weakens in the irrigated croplands highlighting the influence of irrigation. |
|  | **Title:** Spatial and temporal responses of different crop‐growing environments to agricultural drought: a study in Haryana state, India using NOAA AVHRR data  **Authors:** Murthy, C. S., Sesha Sai, M. V. R., Chandrasekar, K., & Roy, P. S.  **Journal:** International Journal of Remote Sensing  **Year:** 2009 | - Analyze the agricultural drought using NDVI data by NOAA‐AVHRR. - Interpreted the results based on the irrigation support information for different districts of Haryana. |
